# Trends and predictive research on the global burden of ischemic heart disease from 1990 to 2021: an analysis of the Global Burden of Disease study 2021

**DOI:** 10.3389/fpubh.2025.1569179

**Published:** 2025-09-19

**Authors:** Pengpeng Liang, Shizhao Zhang, Mei Yan, Hai Huang, Jinhua Kang, Yue Li, Guiyun Li, Hongyan Wu

**Affiliations:** ^1^Shenzhen Hospital, Shanghai University of Traditional Chinese Medicine, Shenzhen, China; ^2^The First School of Clinical Medicine, Jiangxi University of Chinese Medicine, Nanchang, China

**Keywords:** ischemic heart disease, global burden of disease, estimated annual percentage change, risk factor analysis, age-period-cohort, time trend

## Abstract

**Objective:**

To analyze trends in the global burden (GBD) of ischemic heart disease (IHD) over the past 30 years and health inequalities, as well as to predict the trends for the next 25 years.

**Methods:**

Data on the incidence, prevalence, mortality, Disability-Adjusted Life Years (DALYs), and risk factors for IHD were obtained from GBD 2021. Changing global, regional, and national trends from 1990 to 2021 were analyzed, accompanied by decomposition analysis. Potential for improvement was assessed using frontier analysis while conducting a regional risk factor ranking analysis. Joinpoint software and an age-period-cohort model were used to analyze IHD data further. Finally, future IHD trends were analyzed using the prediction models such as the Bayesian age-period-cohort analysis (BAPC) model.

**Results:**

According to GBD 2021, the global incidence of IHD cases was 31,872,778 (201.6%, compared to 1990), with 254,276,268 prevalent cases (226.7%, compared to 1990), 188,360,557 DALYs (158.1%, compared to 1990), and 8,991,637 deaths (167.5%, compared to 1990). Significant changes were noted in Uzbekistan, China, and Indonesia. Male patients outnumbered females, and most were over 60. In 2021, the total amount of IHD was primarily driven by ageing and population growth, with substantial potential for improvement observed in middle and high Socio-Demographic Index (SDI) regions; future attention should also be directed toward occupational risks, air quality, and renal dysfunction’s impact on IHD. The BAPC method showed that the incidence of IHD would reach 56,431,619 cases by 2046. This would be 1.77 times the number of cases in 2021.

**Conclusion:**

From 1990 to 2021, the number of IHD cases in the world and its forecast analysis showed an upward trend, mainly affected by population growth and aging. The disparity of medical burden in areas with low, middle and medium development levels is worsening. The importance of regional rankings of risk factors for IHD has also shifted due to global economic changes over the past 30 years. This study highlights the challenges faced in managing IHD and calls for governments and institutions to adopt multidimensional public health strategies encompassing age, risk factors, gender, and predictive models to address the growing number of cases and other health problems. These findings will guide health policies to effectively control clinical stress in IHD by prioritizing regional risk factors for targeted interventions and individualized prevention for high-risk populations, while also ensuring efficient use of health resources.

## 1 Introduction

Ischemic Heart Disease (IHD), a disease that causes reduced myocardial blood flow and a mismatch between oxygen supply and demand of the myocardium, makes it a major contributor to the burden of cardiovascular diseases. According to the World Health Organization, from 2000 to 2021, IHD is the leading cause of death worldwide, accounting for 13% of all deaths worldwide, and is the second leading cause of Disability Adjusted Life Years (DALY) lost globally, with a significant impact on global public health (1). In 2019, the number of deaths due to IHD in China reached 1.874 million, accounting for about 20.5% of the total number of IHD deaths in the world, and the DALY caused by IHD reached 18.8585 million years, accounting for 10.4% of the total number of Daly induced by IHD in the world (2). In the United States, the economic impact is even more pronounced, with projections showing that total direct medical costs for cardiovascular disease, including IHD, are expected to triple from $273 billion in 2010 to $818 billion in 2030 (3). With the global population aging, dietary and metabolic problems, and the prevalence of obesity-related diseases, the disease burden caused by ischemic heart disease will continue to place enormous pressure on the global health and economic system, and the prevention and control of IHD is urgent.

Gender, as an essential biological and sociological variable, has a significant impact on ischemic heart disease. Studies have shown that the incidence of ischemic heart disease is generally higher in men than in women. Still, over time, women tend to lack the protective mechanism of estrogen after menopause. The risk of IHD increases, especially in women over 70 years old, and the incidence of ischemic heart disease gradually approaches the male level (4, 5). In addition, according to the survey (6), the prevalence and incidence rate of ischemic heart disease peaks in the 55–59 age group. The symptoms of older patients are often not typical, easy to miss a diagnosis, and usually accompanied by other diseases such as hypertension, diabetes, and other chronic diseases, the cause of complex, brutal treatment and surgical risk, easy to develop into heart failure, myocardial infarction, and other serious complications. With the acceleration of the pace of social life and the rapid development of the economy, the incidence of metabolic diseases caused by environmental pollution, work pressure, bad habits, and other factors in the young population has increased year by year, becoming an important driving factor for the trend of ischemic heart disease in the younger population (7). The 2019 GBD on the Burden of Disease in young people aged 15–39 shows an increasing prevalence of ischemic heart disease among young people globally (8). A report indicated that the incidence of acute myocardial infarction (AMI) among young people in the United States increased from 34 per 100,000 in 2016 to 68 per 100,000 in 2020 (9). In the face of the increasingly difficult situation of ischemic heart disease, the hierarchical structure of multi-dimensional characteristics such as age, gender, and education and personalized medical programs should be established to implement precise intervention and treatment strategies and regular health screening to reduce the risk of ischemic heart disease.

However, the current situation is that in most countries around the world, patients with ischemic heart disease do not have access to standardized medical care due to inadequate resources and education in the healthcare system. In low - and middle-income countries in North Africa and the Middle East, resource shortages, combined with inefficient public health awareness and healthcare system effectiveness, severely limit the implementation of effective management and prevention measures for ischemic heart disease (10). This is despite higher relative risk values for CVD risk factors in high-income countries; accidents are lower due to improved healthcare systems, better control of risk factors, and timely and effective treatment measures such as empirical drug regimens and percutaneous coronary intervention (11, 12). This disparity highlights the importance of global collaboration and sharing of experience in tackling the growing global epidemic of ischemic heart disease.

GBD 2021 provides annual disease burden estimates for 369 diseases and injuries and 87 risk factors and combinations across 204 countries and regions from 1990 to 2021. The incidence, prevalence, mortality rates, and DALYs in this database effectively reflect trends in disease severity, offering a unique opportunity to assess the long-term trends of various disease burdens globally, regionally, and nationally. Notably, DALY, one of the summary health indicators, represents the sum of years of life lost (YLL) and years lived with disability (YLD). IHD-related YLL was calculated by multiplying the number of deaths in each age group by the standardized life expectancy for that age group. YLD was calculated by multiplying the prevalence of each mutually exclusive sequela by its disability weight. DALY unifies lethal and non-lethal health impairments into the unit of “health years,” transcending the limitations of traditional mortality statistics and providing a comprehensive depiction of the impact of diseases on population health. At present, studies on the epidemiology of ischemic heart disease are limited in India, North Africa, and the Middle East, and many studies have found that a variety of factors such as elevated residual cholesterol levels, blood sugar levels, autoimmune diseases, smoking, and premature birth are closely related to ischemic heart disease (13–16). Although these studies have analyzed ischemic heart disease from various perspectives, they focus on a country or region, risk factors, specific groups, or overall trends in multiple cardiovascular diseases and lack predictive analysis of time trends. Considering that IHD is a chronic and progressive disease with irreversible structural changes and is the outcome of a variety of diseases with many serious complications, early prevention and treatment are crucial. Therefore, it is necessary to comprehensively describe and analyze the overall incidence and change trend of ischemic heart disease from a global perspective, which is helpful for governments and organizations to take targeted measures and formulate programs to reduce the disease burden of ischemic heart disease. Based on data from the GBD 2021, this article analyzes the disease burden of IHD and its development trends over the next 25 years using key indicators such as incidence rate, prevalence rate, mortality rate, and DALY rate from various aspects for the period 1990–2021.

## 2 Materials and methods

### 2.1 Data source

The GBD 2021 project comprehensively evaluates and publishes the prevalence of 371 diseases and injuries and 88 risk factors across 204 countries and 21 territories worldwide (17). It estimates incidence rates, prevalence rates, mortality rates, years of life lost, healthy life lost, disability-adjusted life years (DALYs), and other disease frequency and burden indicators by gender and age stratification. IHD was defined based on the International Classification of Diseases-Tenth Revision codes I20 to I25.9. This study extracted the estimated number and age-standardized incidence rate, prevalence, mortality, and disability-adjusted life years (DALYs) of IHD and their 95% uncertainty intervals (UIs) from GBD 2021 for the subsequent data analysis. GBD 2021 uses unidentified aggregated data, and the University of Washington’s Institutional Review Board reviewed and approved the waiver of informed consent.

### 2.2 Descriptive analysis

Descriptive analyses were performed at various levels: global, regional, and national, to gain a thorough insight into the impact of ischemic heart disease. The case number and age-standardized rates (ASR, ASIR, ASMR, ASDR) of incidence, prevalence, deaths, and disability-adjusted life years (DALYs) associated with IHD were assessed for 1990 and 2021 on a global scale across 21 geographic areas defined by GBD and in 204 countries and territories and classified by gender. Additionally, comparisons were made within five regions categorized by socio-demographic index (SDI) quintiles. The ASR was computed per 100,000 individuals utilizing the subsequent formula:


$$\mathrm{ASR}=\frac{\sum_{i=1}^{n}(r_{i}\cdot w_{i})}{\sum_{i=1}^{n}w_{i}}\times 100,000$$

($r_{i}$: the age-specific rate in ith the age group; w: the number of people in the corresponding ith age group among the standard population; A: the number of age groups).

### 2.3 Trend analysis

Investigating the temporal patterns of diseases is a fundamental aspect of epidemiology and supports advancing more accurate prevention strategies. The trends in age-standardized rates (ASRs) over a designated time frame are indicated by the estimated annual percentage change (EAPC) (18). The EAPC analysis leverages longitudinal data from all years to capture long-term changes in global disease burden indicators (such as incidence and mortality), avoiding biases from comparing only the first and last years. Standardized methods like age-standardized rates ensure comparability across regions, populations, and time periods, supporting evidence-based decisions for identifying high-risk groups, evaluating interventions, and optimizing resource allocation. The EAPC analysis leverages longitudinal data from all years to capture long-term changes in global disease burden indicators (such as incidence and mortality), avoiding biases from comparing only the first and last years. Standardized methods like age-standardized rates ensure comparability across regions, populations, and periods, supporting evidence-based decisions for identifying high-risk groups, evaluating interventions, and optimizing resource allocation. In short, we employed the regression model y = *α* + βx + *ε* for these analyses, where y represents ln(ASR), x denotes the time variable, and ε signifies the error term. It was assumed that the natural logarithm of ASR has a linear relationship with time; thus, EAPC is calculated as 100 × [exp(*β*) − 1] (Ding et al., 2022). We also utilized linear models to determine the 95% confidence intervals (95% CIs) for EAPCs. An increasing trend in ASR was identified if both the EAPC and its lower bound of the 95% CI > 0. Conversely, an ASR was deemed to exhibit a decreasing trend if both the EAPC and its upper limit of the 95% CI < 0.

### 2.4 Decomposition analysis

Decomposition analysis quantifies the independent contributions of population growth, changes in age structure, and epidemiological variations to the changes in disease burden, revealing underlying causes that a single trend indicator cannot reflect. This provides a scientific basis for precise intervention and policy optimization. Thus, decomposition analysis is employed to recognize the factors linked to the alterations in the absolute quantity of age-related disease burdens (19). It can determine the additive contribution of the effect of the dissimilarities in factors between two groups (populations in 1990 and 2021) to the disparity in their overall disease burden (20). To gain insight into the explanatory factors driving changes in incidence, prevalence, mortality, and DALY rates of IHD from 1990 to 2021, disaggregated analysis was performed by population size, age structure, and epidemiological changes. A detailed method description is provided in the Supplementary methods.

### 2.5 Frontier analysis

In addition to analyzing the interaction of the three factors of IHD in different regions around the world, we also conduct cutting-edge research to assess the relationship between the burden of IHD and the socio-geographical development of various countries and explore the potential for improvement among countries at the same level of development. We used data from 1990 to 2021 to establish a frontier analysis based on incidence, prevalence, mortality, DALYs, and SDI to understand better the potential gaps that a country or region may achieve and the potential for improvement. Supplementary Table S2 offers a detailed account of the frontier analysis methodology (21). A detailed method description is provided in the Supplementary methods.

### 2.6 Risk factor analysis

By reviewing the literature, we found that in addition to traditional factors affecting IHD, the risk factors affecting IHD are also changing with the improvement of social development and environmental changes. We learned from the GBD Database website (GHDx)^1^ query and retrieve SDI, Metabolic factors, behavioral factors, risk factors, dietary risk, high systolic blood pressure, high cholesterol diet, environmental occupational risk, air pollution, particulate matter pollution, tobacco, smoking, low whole grain diet and other risk factors for IHD-related death and DALYs in five regions of the world from 1990 to 2021 by sex (total, male and female). Based on the relative size of the values in the downloaded data table, rankings were established, and a heat map illustrating the areas categorized by risk factors and the Socio-Demographic Index (SDI) was created using the R package “heatmap.”

### 2.7 Joinpoint analysis

Joinpoint regression analysis has become a core tool for analyzing complex epidemiological patterns due to its ability to accurately identify turning points of trends and quantify dynamic changes at different stages. So, the joinpoint regression analysis was employed to evaluate the epidemiologic data of IHD over the study period. This method segments the timeline into distinct intervals, each featuring a separate regression line, allowing for a nuanced evaluation of trend changes over time (22). This approach outperforms traditional regression models by capturing localized trend fluctuations. It provides an objective basis for phased adjustments of prevention and control strategies across periods, enhancing the interpretation of disease dynamics and the timeliness of policy interventions. We employed the software provided by the United States National Cancer Institute (version4.9.1.0, 2022) for the analysis, focusing on key indicators including annual percentage change (APC), average annual percentage change (AAPC), and 95% confidence intervals (95% CI). Moreover, the average APC (AAPC) for multiple-year periods was estimated using regression coefficients weighted by the span width of each subsegment. The formula of the joinpoint regression model is as follows:


$$\begin{array}{rr} & {\mathrm{APC}}_{i}=\{\exp(\mathrm{\beta i})-1\}\times 100\%\\ & {\text{AAPC}}_{i}=\{\exp(\frac{\sum W_{i}\beta_{i}}{\sum W_{i}})-1\}\times 100\% \end{array}$$

The number and location of joinpoints were determined using the Grid Search Method; in the model, i is the number of segments, βi corresponds to the regression coefficients for each linear segment of the data, and Wi represents the length of each corresponding segment. The Monte Carlo ranking method was used to calculate AAPC and 95% CI, and the overall asymptotic significance level was maintained by Bonferroni correction (23). If the APC/AAPC estimate and the lower bound of its 95% CI are greater than 0, an upward trend is considered over a specific period. Conversely, if the estimated APC/AAPC value and the upper bound of its 95% CI are less than 0, a downward trend is regarded within a specific period. Otherwise, the trend is considered stable.

### 2.8 Age–period–cohort analysis

We conducted an in-depth investigation into the trends of the burden of ischemic heart disease and its effects on age, period, and birth cohort. We initially inspected the potential two-factor interactions among age, period, and birth cohorts using Stata to achieve this objective. Due to the interaction between age, period, and birth cohort, it proves challenging to determine their actual influence on the risk of onset or epidemic. An age-period-cohort model adopting the intrinsic estimator (IE) approach is employed to solve this problem (24, 25). The age effect clarifies factors such as the impact of aging populations on morbidity and mortality rates. The period effect refers to changes in the risk of diseases and injuries due to objective factors. The cohort effect indicates that different generations have varying levels of exposure to disease risk factors. The APC model can generally be represented as: Y = log (M) = *μ* + *α*(age)i + *β*(period)j + *γ*(cohort)k + *ε*. In this equation, M represents the age-standardized rate (asr) of IHD, μ, and ε denote the intercept and random error, respectively. In contrast, *α*(age)i, *β*(period)j, and *γ*(cohort)k represent the effects of age group α, period β, and birth cohort γ. The method furnishes estimated coefficients for age, period, and birth cohort effects. Subsequently, the coefficients are converted into index values to ascertain the relative risk of a specific age, period, or birth cohort epidemiological indicator when compared with all ages, periods, or birth cohorts (26). The study used 17 consecutive 5-year age intervals, ranging from 0–4 years to 95–99 years; it also analyzed six-period effects from 1992–1996 (median 1994) to 2017–2021 (median 2019). Regarding cohort effects, the 30-year study period included data from 26 consecutive birth cohorts, from 1897–1901 (the 1899 cohort) to 2002–2006 (the 2004 cohort).

### 2.9 Predictive analysis

Through these analyses, we have a preliminary understanding of the changes in ischemic heart disease in the past three decades, and further projections of the burden of ischemic heart disease in the next 25 years are made to formulate public health policies better and allocate medical resources. BAPC forecasting is a Bayesian statistical analysis technique used to predict future events or trends. BAPC forecasting effectively decomposes and estimates age, period, and cohort effects, providing predictions of future events and quantifying uncertainty, while also being flexible in handling complex data structures. Bayesian Age-period-cohort analysis (BAPC) models with integrated nested Laplacian approximations (INLA), with better coverage and accuracy than APC models, were used to predict the global burden of ischemic heart disease over the next 25 years (27). The model structure is a linear Poisson model, which assumes the multiplicative effects of age, period, and cohort variables. The formula for the BAPC model is:


$$\log(\lambda_{\mathrm{ij}})=\alpha +\mu_{i}+\beta_{j}+\gamma_{k}$$

In this model, i(1 < i < D) represents time points, j(1 < j < J) denotes age groups, a represents the intercept, μi represents the age effect, ßj represents the period effect, γk represents the cohort effect. We also used the Nordpred package in R software to analyze trends in ischemic heart disease, calculating ASR and patient cases by sex (28). It is a log-linear age-period-cohort model designed to predict the number or rate of new cases, which can mitigate exponential growth and restrict linear trend predictions to accommodate recent trends, demonstrating its effectiveness in forecasting future burden trends. The differential autoregressive moving average (ARIMA) model in the time series model is also used to predict the future trend of ischemic heart disease (29). The ARIMA model integrates autoregression (AR), differencing (I), and moving average (MA) to transform non-stationary time series into stationary ones through differencing, while capturing the autocorrelation of the time series using AR and MA to forecast future trends. The ARIMA model requires that the time series be a random sequence with a mean of zero and exhibit stationarity. Finally, the development trend of IHD in the future is evaluated reasonably by using these three methods.

## 3 Results

### 3.1 Descriptive analysis of the burden of IHD at global, regional and country levels

Globally, the case numbers of incidence, prevalence, and DALYs for ischemic heart disease patients have increased. The incidence of cases increased from 15,813,619 (13180529–18,849,479) in 1990 to 31,872,778 (26284921–38,267,834) in 2021 (201.6%). In terms of prevalence, the number of patients increased from 112,169,488 (99416741–125,730,169) in 1990 to 254,276,268 (221446458–295,493,093) in 2021 (226.7%). DALYS changed from 119,162,957 (114547787–123,454,733) in 1990 to 188,360,557 (177036930–198,154,477) in 2021 (158.1%). The number of deaths increased from 5367136.581 (5076403.864–5562773.874) to 8,991,637 (8264123–9,531,130) in 2021 (167.5%). In addition, ASIR declined globally from 419.54 (351.07, 498.17) per 100,000 people in 1990 to 372.9 per 100,000 people in 2021 (Supplementary Table S8). The ASPR increased from 2904.72 per 100,000 people in 1990 to 2946.38 per 100,000 people in 2021 (Supplementary Table S9). ASDR rose from 158.9 per 100,000 people in 1990 to 108.73 per 100,000 people in 2021 (Supplementary Table S10). ASMR dropped from 3,107.61 per 100,000 people in 1990 to 2,212.16 per 100,000 people in 2021 (Supplementary Table S11). We next further captured changes in age-standardized rates of IHD epidemiological measures using the EAPC method.

### 3.2 Global trend analysis of IHD at global, regional and country levels

**Figure 1:**
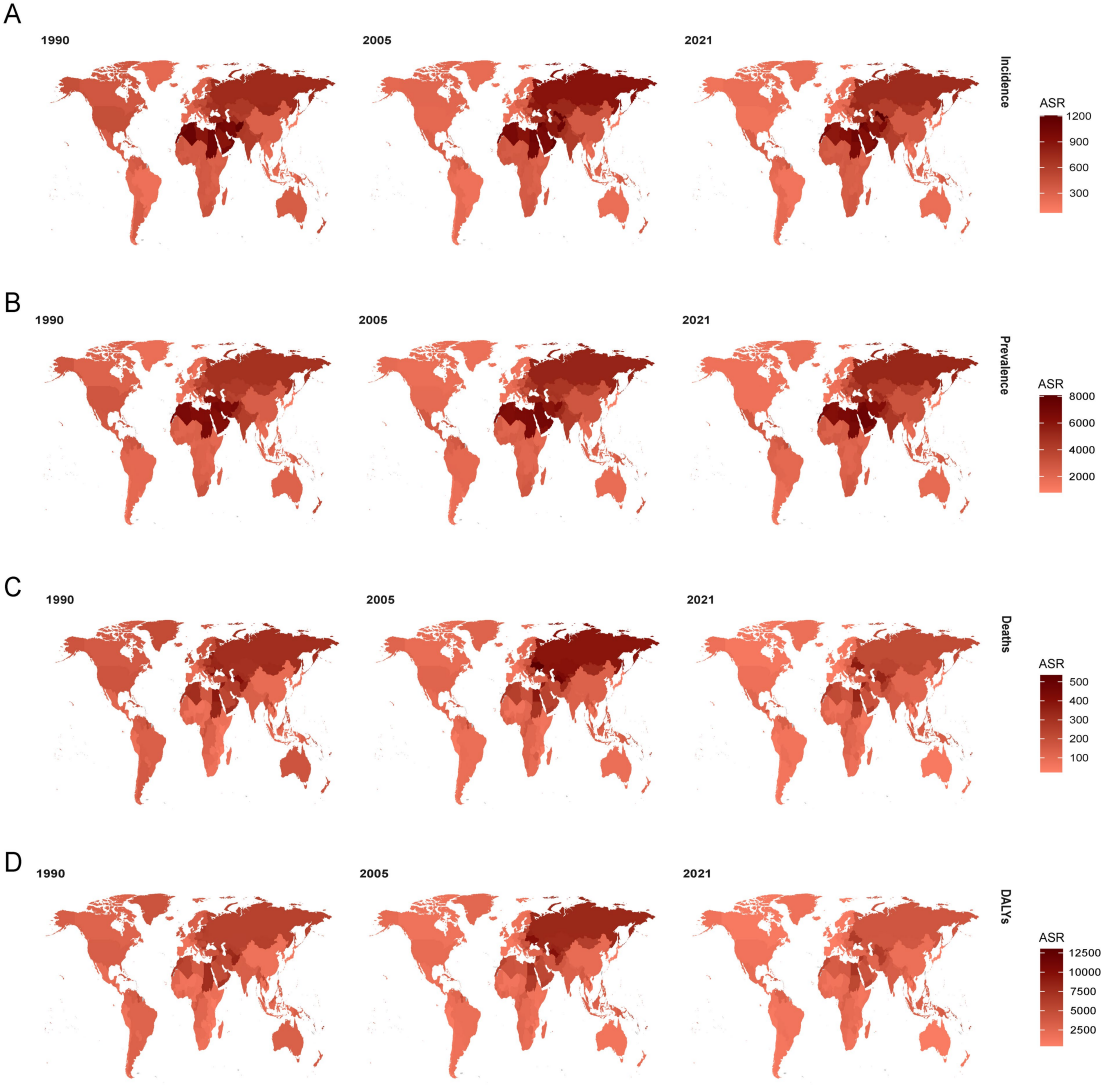
**(A)** The ASR of incidence in 1990, 2005, 2021. **(B)** The ASR of prevalence in 1990, 2005, 2021. **(C)** The ASR of deaths in 1990, 2005, 2021. **(D)** The ASR of DALYs in 1990, 2005, 2021. IHD, Ischemic heart disease, DALYs, disability-adjusted life-years.

**Figure 2:**
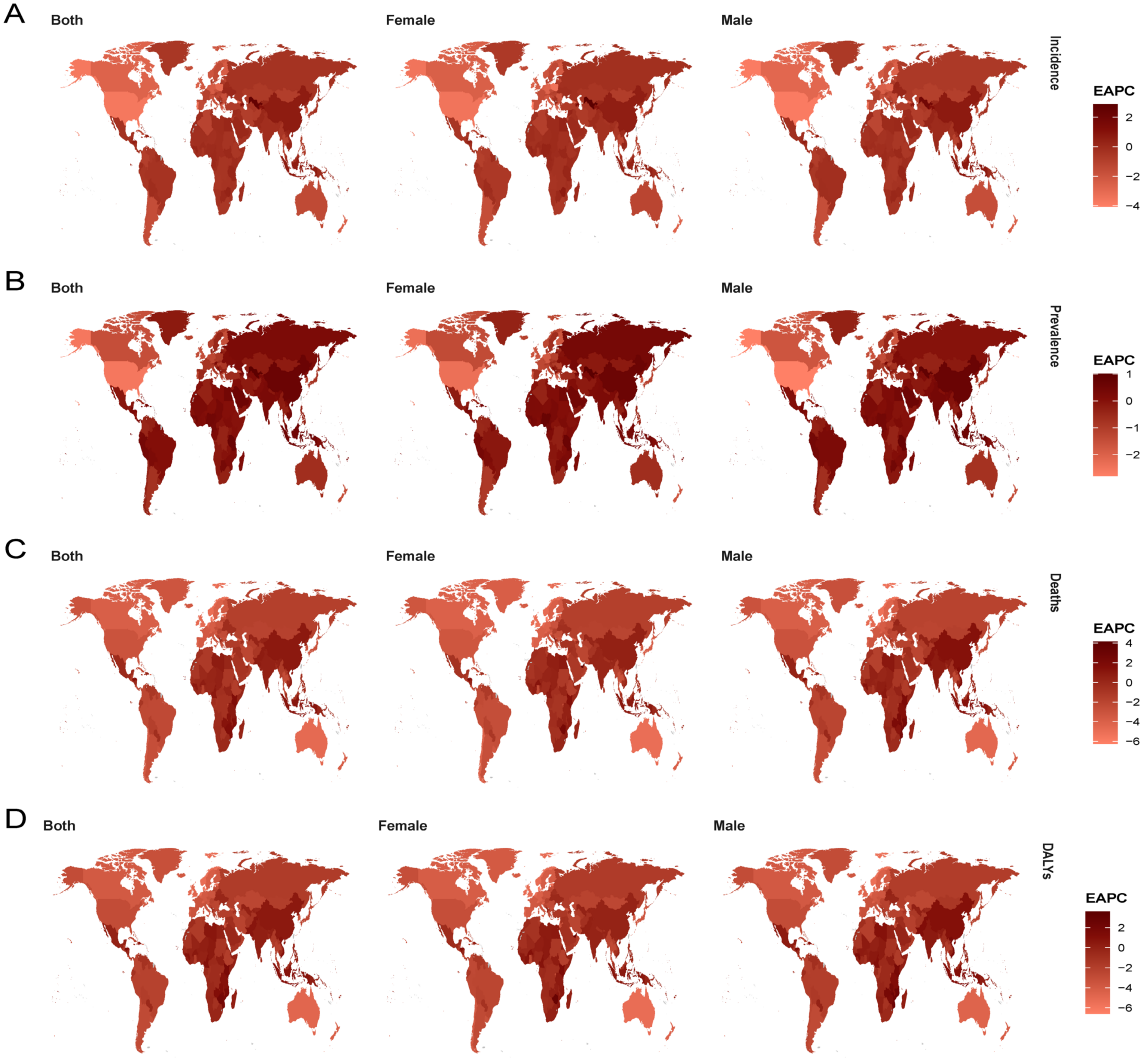
**(A)** The trend in ASR of incidence (EAPC) from 1990 to 2021; **(B)** The trend in ASR of prevalence (EAPC) from 1990 to 2021; **(C)** The trend in ASR of deaths (EAPC) from 1990 to 2021; **(D)** The trend in ASR of deaths (EAPC) from 1990 to 2021. IHD, Ischemic heart disease, DALYs, disability-adjusted life-years, EAPC, estimated annual percentage change.

Globally, the ASIR for ischemic heart disease has decreased from 1990 to 2021. However, regional trends reveal an increase in ASIR in Central Asia (EAPC, 0.7), East Asia (EAPC, 0.621), and Oceania (EAPC, 0.035) during the same period (Supplementary Table S8). The ASPR has shown a steady upward trend from 1990 to 2021, with significant increases observed in Latin America (EAPC, 0.342), Central Asia (EAPC, 0.237), East Asia (EAPC, 0.614), Eastern Europe (EAPC, 0.204), East Sub-Saharan Africa (EAPC, 0.116), Oceania (EAPC, 0.145), South Asia (EAPC, 0.218), and Southeast Asia (EAPC, 0.166; Supplementary Table S9). Notably, the DALYs for ischemic heart disease have increased in East Asia (EAPC, 0.465), South Asia (EAPC, 0.165), and Sub-Saharan Africa (EAPC, 0.107), contrary to the global trend of declining DALYs (Supplementary Table S11). Moreover, while mortality rates have generally declined in most regions of the world, there has been an increase in death rates in East Asia (EAPC, 0.896), South Asia (EAPC, 0.436), Sub-Saharan Africa (EAPC, 0.266), and West Sub-Saharan Africa (EAPC, 0.024) over the past 30 years (Supplementary Table S10). At the national level, Turkmenistan, Russian Federation, Ukraine, Azerbaijan, Yemen, Afghanistan, Sudan, Morocco, Iraq, Egypt, Syrian Arab Republic, and Uzbekistan consistently rank among the highest in terms of ASR of incidence, prevalence, mortality, and DALYs of IHD in 2021. Portugal, Japan, Chile, Luxembourg, Greece, United Kingdom, Spain, Switzerland, Singapore, Andorra, Norway, Ireland, United States of America, France, and the Netherlands consistently rank among the lowest in terms of incidence, prevalence, mortality, and DALYs of IHD in 2021 (Figure 1; Supplementary Table S1). The highest EAPC of epidemiological indicators of IHD are found in Uzbekistan, China, Indonesia, the Dominican Republic, Honduras, Vietnam, Timor-Leste, Lesotho, the United Republic of Tanzania, Guinea, Djibouti, Eswatini, Egypt, Cape Verde, and Cameroon. Conversely, Portugal, Georgia, Canada, Ireland, New Zealand, Belgium, Germany, the Netherlands, Israel, the United Kingdom, the United States, Norway, Iceland, Australia, Switzerland, Slovenia, and Cyprus exhibit the lowest EAPC values for IHD. According to the classification of regions by the SDI quintiles over the past three decades, the burden in most areas, such as high-SDI regions and high-middle SDI regions, has exhibited a downward trend in terms of epidemiological indicators of IHD (Figure 2; Supplementary Table S2). In contrast, the burdens of incidence and prevalence have slightly increased in middle SDI regions, while the burdens of prevalence, mortality, and disability-adjusted life years (DALYs) have also increased in low-middle SDI regions. Additionally, the burden has slightly increased within low SDI regions (Supplementary Tables S8–S11).

### 3.3 Analysis of global disease age accumulation map

**Figure 3:**
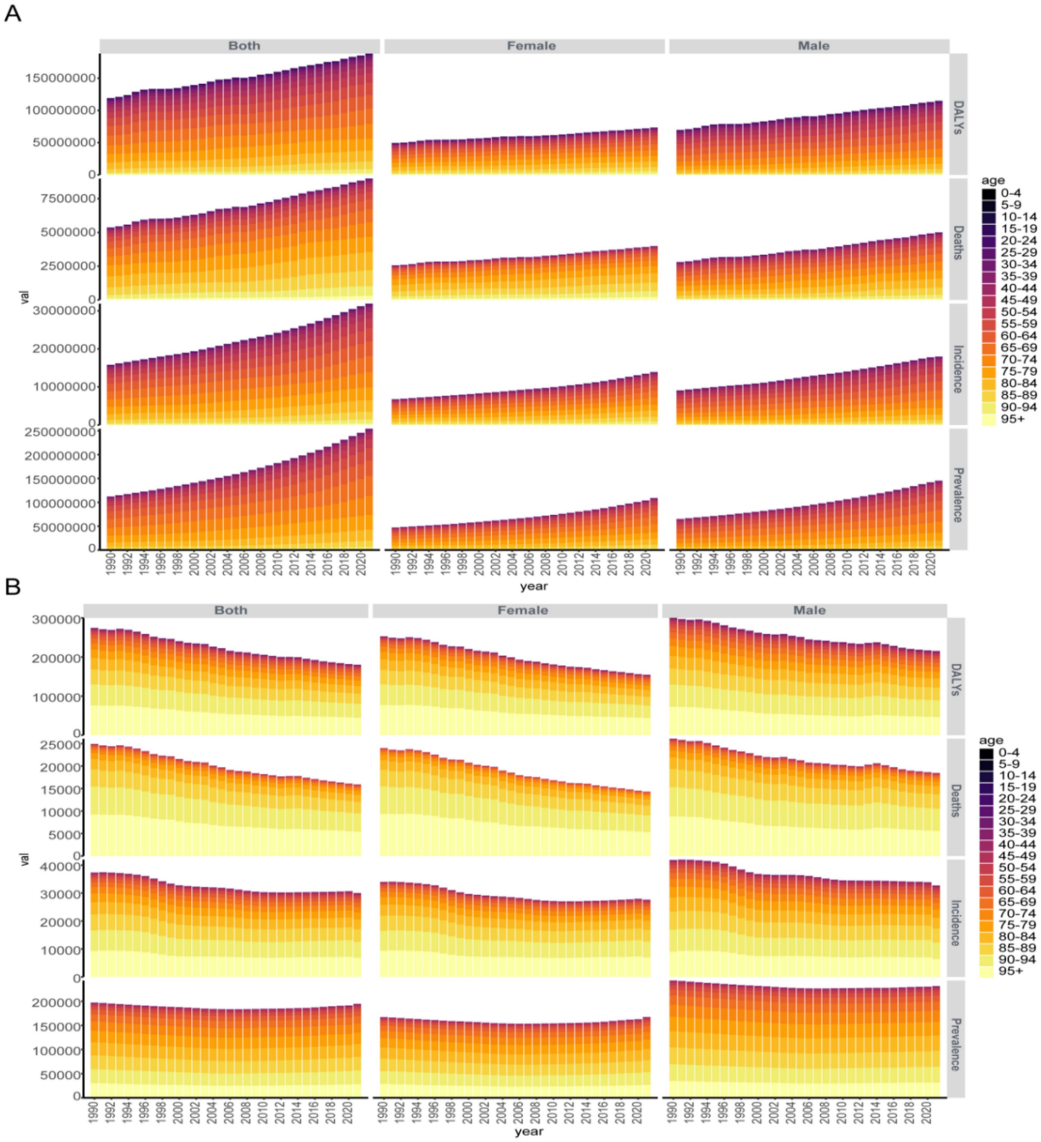
**(A)** The global age accumulation map of case number of IHD from 1990 to 2021. **(B)** The global age accumulation map of ASR of IHD from 1990 to 2021.

By mapping IHD changes across regions and countries, we gained an initial understanding of the overall trend. To further examine the age distribution of IHD over time and support future targeted interventions, we created age-stacked bar charts for the disease. As depicted in Figure 3, the global numbers of incidence, prevalence, mortality, and DALYs of IHD have increased with the passing years. Among them, the accumulated number of male patients each year is far greater than that of female patients. The age groups of 60–64, 65–69, 70–74, 75–79, and 80–84 have relatively larger numbers. Regarding the ASR, the global numbers of incidence, prevalence, mortality, and DALYs of IHD have decreased over the years. Similarly, the accumulated values of male patients each year are much higher than those of female patients, among which 85–89 years old, 90–94 years old, and 95 + age group accounted for the major part of all age groups.

### 3.4 Decomposition analysis

**Figure 4:**
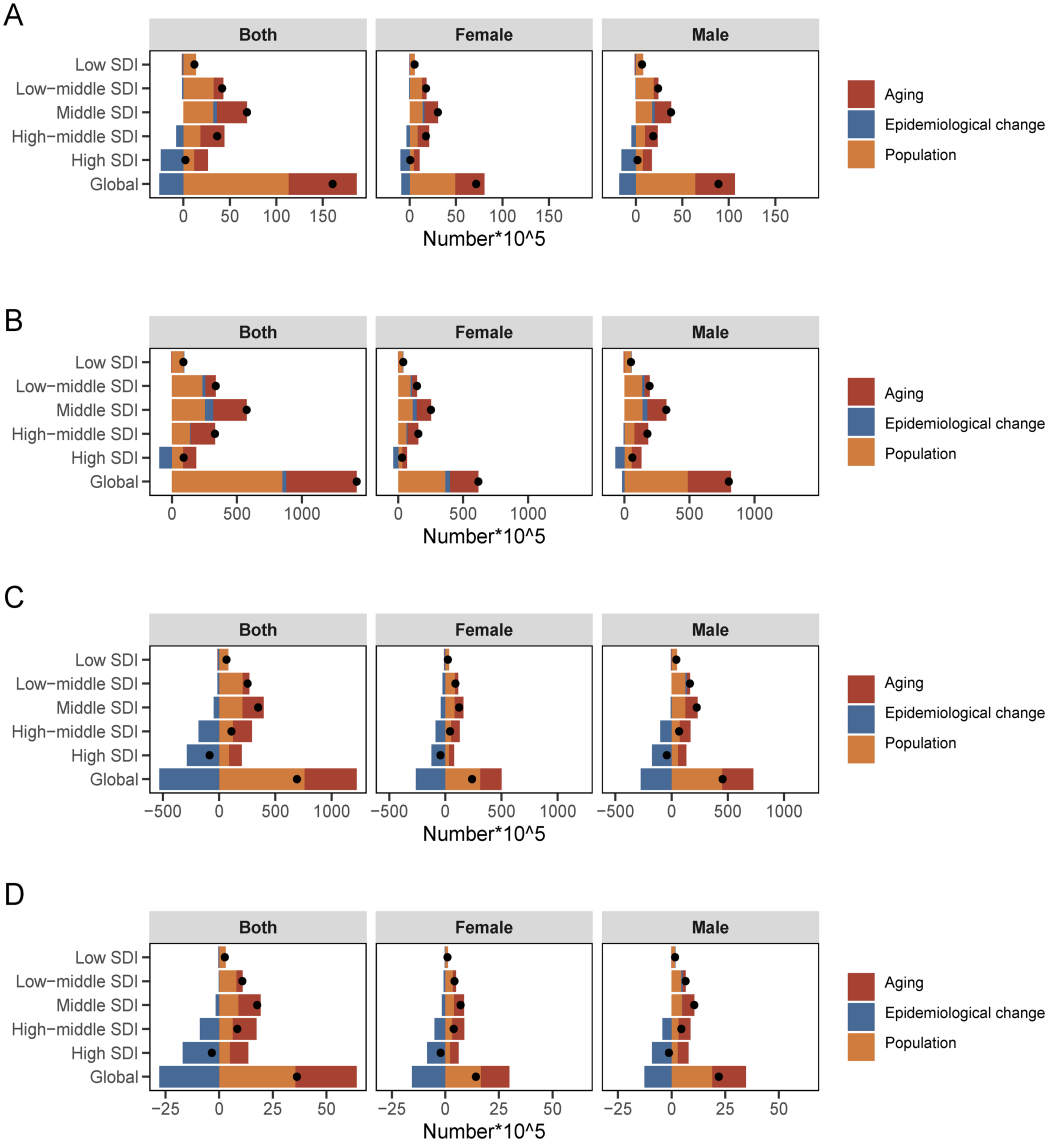
Changes in the incidence **(A)**, prevalence **(B)**, DALYs **(C)**, deaths **(D)** of IHD according to aging, population growth and epidemiological change from 1990 to 2021 at global level by SDI quintile and by subgroups of sexes.

To further understand the specific contributions of age structure, population growth, and epidemiological changes to IHD, we disaggregated the five regions divided by SDI and the global IHD data. In 2021, there were 31.87 million incidence cases of IHD worldwide, with 254.27 million cases of prevalence for IHD, which were 201.55 and 226.69% of the data from 1990, respectively. Additionally, in 2021, the number of DALYs and deaths caused by ischemic heart disease were 18.836 million and 5.36 million, which were 158.07 and 59.69% of the data from 1990, respectively. Globally and in most regions, according to SDI, the increase in ischemic heart disease is mainly attributed to population growth and aging. However, in high and middle SDI regions, the impact of aging on incidence (671.69, 71.14%), prevalence (113.11, 56.55%), DALYs (136.1, 154.37%), and mortality (253.46, 131.4%) is more significant. For patients with ischemic heart disease in low SDI regions, the contribution of population growth to incidence, prevalence, DALYs, and mortality is more prominent, respectively, at 113.76, 104.44, 123.1, and 115.44%. Epidemiological changes have a negative overall contribution to the incidence, DALYs, and mortality rates globally and in the five regions according to SDI quintiles and a relatively more minor contribution to the prevalence rates in the five regions (−2.05, −1.33%, −108.15, 6.44, 2.57, 10.77%). Overall, the drivers of ischemic heart disease globally and in most SDI regions are driven by aging and population growth. Moreover, according to the decomposition results, the impact of the three major factors on male and female groups in different regions is significantly different, and the impact on male patients is higher than that on female patients (Figure 4; Supplementary Table S3).

### 3.5 Frontier analysis

**Figure 5:**
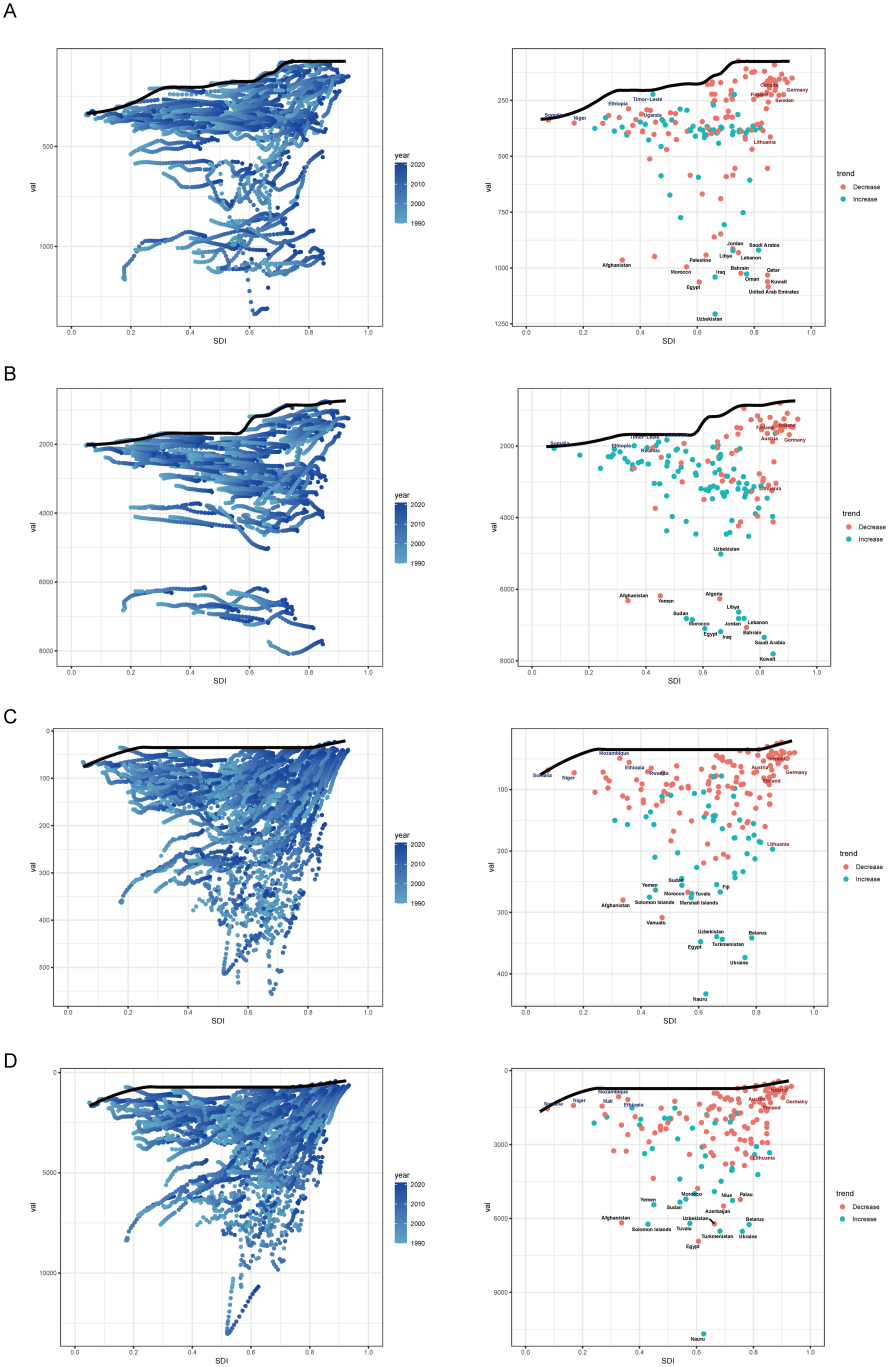
**(A)** Frontier analysis based on SDI and Ischemic heart disease incidence rate from 1990 to 2021 and 2021. **(B)** Frontier analysis based on SDI and Ischemic heart disease prevalence rate from 1990 to 2021 and 2021. **(C)** Frontier analysis based on SDI and Ischemic heart disease death rate from 1990 to 2021 and 2021. **(D)** Frontier analysis based on SDI and Ischemic heart disease DALYs rate from 1990 to 2021 and 2021. The frontier is delineated in solid black color.

We conducted a frontier analysis using IHD Data, including ASIR, ASPR, ASMR, and ASDR data from 1990 to 2021 and the SDI data from 2021 to better understand a country’s potential for improving its situation. (Figure 5; Supplementary Table S4). The boundaries represent the countries and regions with the lowest disease burden at a given SDI level. The actual difference is the distance from the borderline, representing the difference between a country’s actual burden and the minimum burden that can be achieved based on its level of development. Dots represent countries and regions, blue dots indicate upward trends, and red dots indicate opposite trends. Overall, with the development of social demography in 2021, the practical differences between countries have increased to a certain extent. Regarding ASIR, The top 10 countries or regions with the most significant difference in effectiveness from the frontier include Uzbekistan, United Arab Emirates, Kuwait, Qatar, Syrian Arab Republic, Oman, Bahrain, Iraq, Egypt, and Lebanon. The top 10 differences between ASPR and frontier values are Kuwait, Saudi Arabia, Qatar, Bahrain, Iraq, Oman, Lebanon, Jordan, Egypt, Libya, etc. In terms of ASMR, the top 10 countries or regions with the most significant difference in effectiveness from the frontier include Nauru, Ukraine, Syrian Arab Republic, Egypt, Turkmenistan, Belarus, Uzbekistan, Vanuatu, Azerbaijan, Afghanistan. In terms of ASDR, The top 10 countries or regions with the most significant difference in effectiveness from the frontier include Nauru, Vanuatu, Egypt, Syrian Arab Republic, Ukraine, Marshall Islands, Turkmenistan, Belarus, Micronesia Federated States of America, Solomon Islands. It indicates that countries or regions in the middle and high SDI regions have greater improvement potential. Subsequently, we conducted a risk factor analysis for different SDI regions and analyzed the ranking changes of IHD risk factors over 30 years.

### 3.6 Heatmap of risk factor analysis

**Figure 6:**
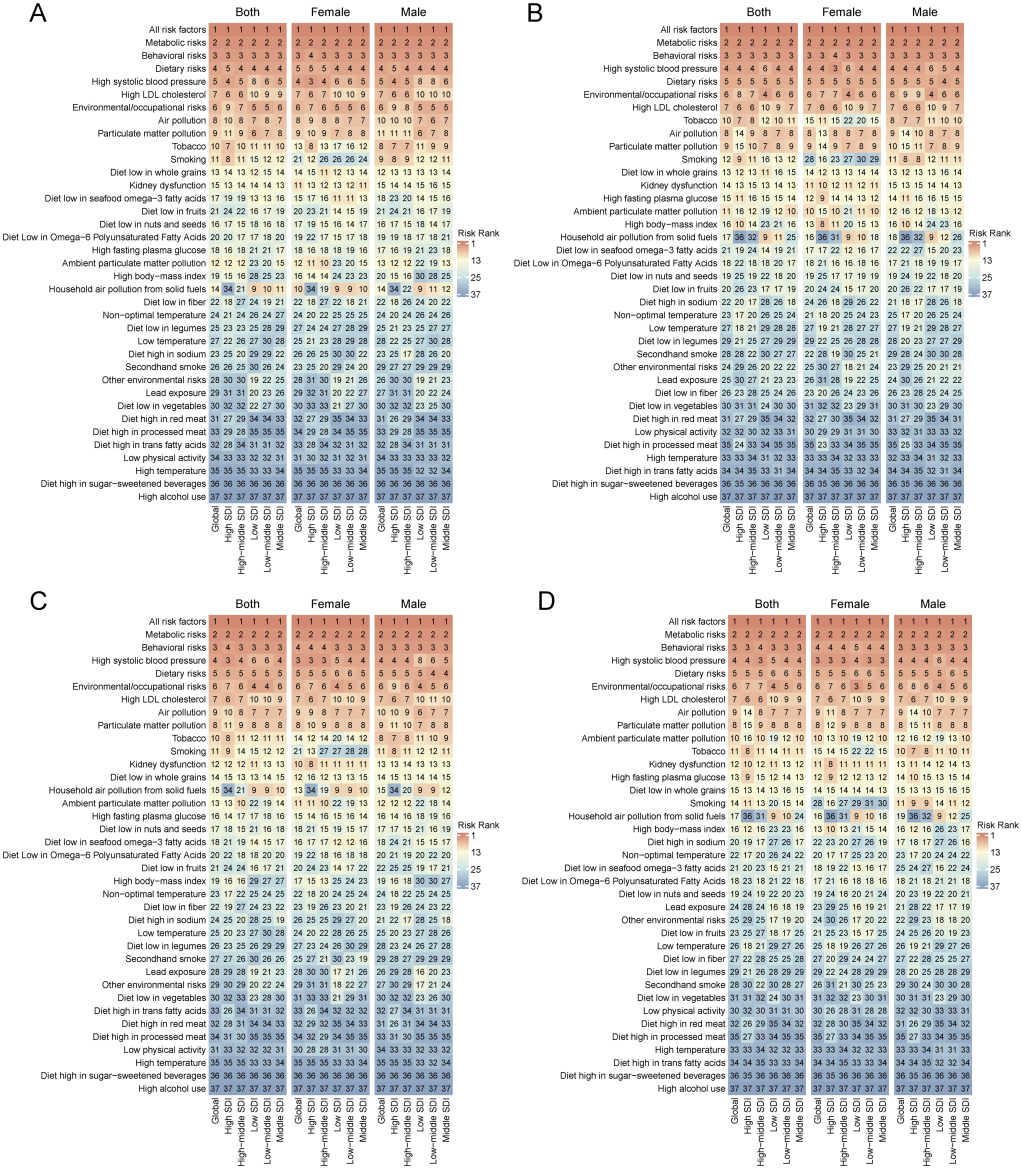
Analysis of risk factors of DALYs and deaths for IHD by global and SDI quintile regions in 1990 **(A,C)** and 2021 **(B,D)**.

In 1990, the top five risk factors (except for “all risk factors”) leading to death and disability in IHD patients were metabolic risks, behavioral risks, dietary risks, high systolic blood pressure, and environmental and occupational risks. In high-SDI and high-middle SDI regions, a high-cholesterol diet has supplanted occupational and ecological risks as a significant cause of death among IHD patients, further accentuating regional characteristics. Among the factors that contribute to IHD deaths in low-income SDI regions, the ranking of particulate pollution is ascending gradually. When comparing the heat maps of risk factors categorized by gender, region, and across the years 1990 and 2021, we can discern that women in high SDI areas are more likely to be affected by blood pressure. In high school SDI areas, women are more at risk than men in environmental occupations. In low-SDI and low-middle SDI areas, particulate matter pollution and air pollution are important risk factors for the death and disability of male patients with IHD. By 2021, the significance of the top five risk factors in 1990 will exhibit less fluctuation across most regions. Environmental/occupational risks in areas with low SDI assume an increasingly crucial role in causing death and disability related to IHD. Gender has little effect on the difference in risk factors in different regions. Over the past three decades, the primary risk factors for ischemic heart disease, ranking sixth to tenth among all global populations, mainly consist of a high-cholesterol diet, air pollution, particulate matter pollution, tobacco use, smoking, and environmental particulate matter pollution. We found that renal dysfunction ranks as an emerging risk factor among the important risk factors for ischemic heart disease disability. In the future, government agencies and doctors should pay attention to the risk of ischemic heart disease in addition to individual diet, behavior, and metabolic risk, but also to ambient air quality and renal dysfunction (Figure 6).

### 3.7 Joinpoint analysis

**Figure 7:**
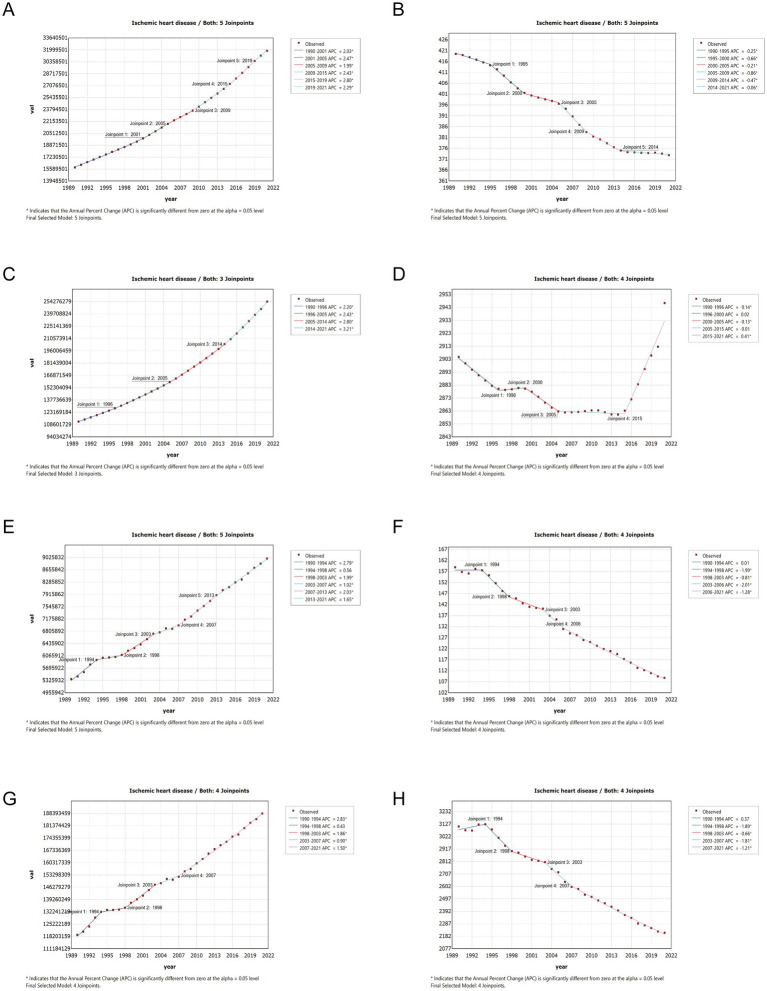
**(A)** The joinpoint regression analysis on the case number of incidence; **(B)** the joinpoint regression analysis on the ASR of incidence; **(C)** the joinpoint regression analysis on the case number of prevalence; **(D)** the joinpoint regression analysis on the ASR of prevalence; **(E)** the joinpoint regression analysis on the case number of deaths; **(F)** the joinpoint regression analysis on the ASR of deaths; **(G)** the joinpoint regression analysis on the case number of DALYs; **(H)** the joinpoint regression analysis on the ASR of DALYs; of IHD globally. ASR, age-standardized rate; DALYs, disability-adjusted life-years.

We further conducted a joinpoint analysis to capture the key change points and trends at each stage of the epidemiological data of IHD over nearly 30 years, enhance the interpretation of disease dynamics, and provide the basis for future policy adjustments. The node regression analysis results of the IHD load are shown in Figure 7. From 1990 to 2021, the consecutive case numbers of incidence, prevalence, mortality rate, and DALYs for IHD showed a marked upward trend, accompanied by the four prominent ascending cycles of 2015–2019, 2005–2014, 1990–1994, and 1990–1994, which, respectively, had 5, 3, 5 and 4 joinpoints. For ASR, the trends in incidence, prevalence, mortality, and DALYs over the 1990–2021 period differed by node segmentation. Specifically, the incidence decreased significantly from 1995–2000, 2005–2009, 2009–2014, followed by 1990–1995, 2000–2005, and slightly from 2014 to 2021. Compared with other epidemiological indicators, the prevalence rate showed a downward trend followed by an upward trend. It decreased significantly from 1990 to 1996 and 1998 to 2003 and increased dramatically from 2015 to 2021, with no significant change in other years. The mortality rate showed a decreasing trend, with no substantial change from 1990 to 1994, and a significant decrease from 1994 to 1998, 2003 to 2006, followed by 1998 to 2003, 2006 to 2021. The DALYs showed a trend of first increasing and then decreasing, with no change in 1990–1994 and a significant decrease in 1998–2003, followed by 1994–1998, 2003–2007, 2007–2021.

### 3.8 Age-period-cohort analysis

**Figure 8:**
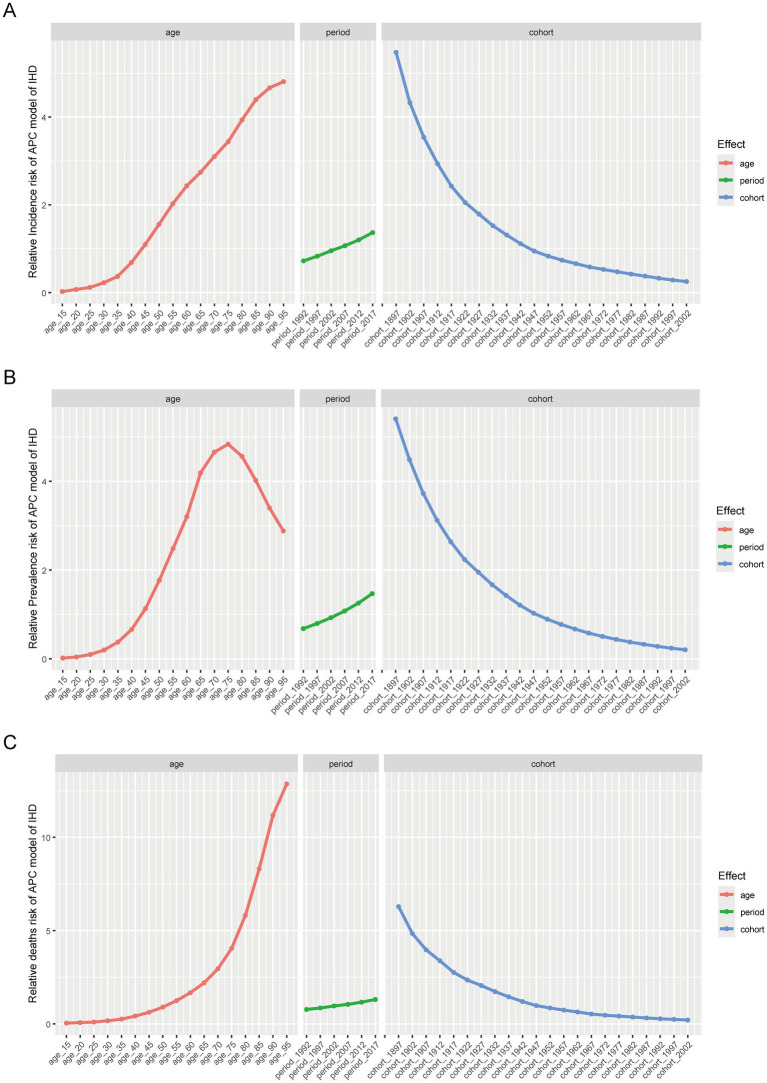
The effects of age, period, and birth cohort on the relative risk of IHD incidence **(A)**, prevalence **(B)** and deaths **(C)**. IHD, Ischemic heart disease.

We first assessed the effects of any two variables in the patient’s age, period, and birth cohort on the epidemiology of ischemic heart disease (Supplementary Figure S1). To address the issue of collinearity among age, period, and cohort, we further employed an age-period-cohort model based on the IE (intrinsic estimator) method to estimate the impact of these factors on the epidemiological trend of IHD. Overall, the APC model analysis shows that ischemic heart disease is affected by age, period, and birth cohort effects, with statistical significance. In the age effect, the incidence and mortality rates of ischemic heart disease generally increase with age, while the prevalence rate shows an upward trend initially and then decreases. Among them, the incidence risk, prevalence risk, and mortality risk for the 15–19 age group were the lowest, with risk values of 0.023 (95%CI, 0.023–0.023), 0.018 (95%CI, 0.018–0.018), and 0.041 (95%CI, 0.040–0.041), respectively. The highest risk of morbidity and mortality was found at age 95 and older, with risk values of 4.808 (4.802–4.815) and 12.864 (12.843–12.885), respectively. The prevalence risk of ischemic heart disease peaked at age 75–79, with a risk value of 3.44 (3.438–3.442), according to model fitting. In the period effect, the incidence risk, prevalence risk, and mortality risk of IHD patients have all shown an upward trend from 1990 to 2021, and the maximum and minimum RR appeared in the sixth-period group (2017–2021) and the first-period group (1992–1996). The former’s incidence, prevalence, and mortality risk are 1.89 times, 2.16 times, and 1.70 times the latter. In the cohort-effect coefficients, the risk of incidence, prevalence, and death of patients with ischemic heart disease decreased with the birth cohort, and the risk values for those born between 1897 and 1901 were 5.476 (95%CI, 5.455–5.497), 5.405 (95%CI, 5.394–5.417), 6.286 (95%CI, 6.260–6.311). The risk values for births from 2002 to 2006 were 0.251 (95%CI, 0.249–0.254), 0.205 (95%CI, 0.204–0.206), and 0.203 (95%CI, 0.2–0.206). The former’s incidence, prevalence, and death were 21.82 times, 26.37 times, and 30.97 times of the latter, respectively (Figure 8; Table 1).

**Table 1:** RRs of IHD incidence, prevalence, and deaths for both sexes due to age, period, and birth cohort effects.

Factor	Incidence		Prevalence		Deaths	
	RR(95CI)	*p*	RR(95CI)	*p*	RR(95CI)	*p*
Age (years)						
15–19	0.023(0.023–0.023)	<0.001	0.018(0.018–0.018)	<0.001	0.041(0.04–0.041)	<0.001
20–24	0.07(0.07–0.071)	<0.001	0.042(0.042–0.042)	<0.001	0.068(0.068–0.068)	<0.001
25–29	0.116(0.116–0.117)	<0.001	0.098(0.098–0.098)	<0.001	0.098(0.098–0.099)	<0.001
30–34	0.223(0.223–0.223)	<0.001	0.2(0.2–0.2)	<0.001	0.165(0.165–0.166)	<0.001
35–39	0.367(0.367–0.368)	<0.001	0.377(0.377–0.378)	<0.001	0.254(0.254–0.255)	<0.001
40–44	0.685(0.684–0.685)	<0.001	0.662(0.661–0.662)	<0.001	0.416(0.415–0.417)	<0.001
45–49	1.095(1.095–1.096)	<0.001	1.13(1.13–1.131)	<0.001	0.62(0.619–0.621)	<0.001
50–54	1.556(1.555–1.557)	<0.001	1.767(1.766–1.767)	<0.001	0.898(0.897–0.899)	<0.001
55–59	2.027(2.026–2.029)	<0.001	2.483(2.483–2.484)	<0.001	1.245(1.243–1.246)	<0.001
60–64	2.432(2.43–2.433)	<0.001	3.202(3.202–3.203)	<0.001	1.669(1.668–1.671)	<0.001
65–69	2.743(2.742–2.745)	<0.001	4.191(4.19–4.192)	<0.001	2.199(2.197–2.201)	<0.001
70–74	3.101(3.099–3.102)	<0.001	4.658(4.657–4.659)	<0.001	2.95(2.947–2.952)	<0.001
75–79	3.44(3.438–3.442)	<0.001	4.835(4.834–4.836)	<0.001	4.047(4.044–4.051)	<0.001
80–84	3.937(3.935–3.939)	<0.001	4.562(4.561–4.564)	<0.001	5.817(5.812–5.821)	<0.001
85–89	4.399(4.396–4.401)	<0.001	4.02(4.019–4.021)	<0.001	8.309(8.301–8.317)	<0.001
90–94	4.67(4.667–4.674)	<0.001	3.399(3.398–3.4)	<0.001	11.179(11.166–11.192)	<0.001
95 plus	4.808(4.802–4.815)	<0.001	2.882(2.88–2.884)	<0.001	12.864(12.843–12.885)	<0.001
Period						
1992	0.722(0.722–0.723)	<0.001	0.681(0.681–0.681)	<0.001	0.771(0.77–0.771)	<0.001
1997	0.829(0.829–0.829)	<0.001	0.796(0.796–0.796)	<0.001	0.852(0.852–0.853)	<0.001
2002	0.952(0.952–0.953)	<0.001	0.926(0.926–0.927)	<0.001	0.958(0.958–0.959)	<0.001
2007	1.068(1.067–1.068)	<0.001	1.079(1.079–1.079)	<0.001	1.046(1.045–1.046)	<0.001
2012	1.201(1.2–1.201)	<0.001	1.256(1.256–1.256)	<0.001	1.163(1.162–1.163)	<0.001
2017	1.368(1.367–1.368)	<0.001	1.469(1.469–1.47)	<0.001	1.307(1.306–1.308)	<0.001
Birth cohort						
1897–1901	5.476(5.455–5.497)	<0.001	5.405(5.394–5.417)	<0.001	6.286(6.26–6.311)	<0.001
1902–1906	4.326(4.318–4.335)	<0.001	4.486(4.482–4.491)	<0.001	4.843(4.832–4.854)	<0.001
1907–1911	3.543(3.538–3.547)	<0.001	3.727(3.724–3.729)	<0.001	3.97(3.964–3.977)	<0.001
1912–1916	2.938(2.935–2.941)	<0.001	3.121(3.119–3.122)	<0.001	3.38(3.376–3.385)	<0.001
1917–1921	2.426(2.424–2.428)	<0.001	2.636(2.635–2.637)	<0.001	2.757(2.754–2.761)	<0.001
1922–1926	2.052(2.051–2.054)	<0.001	2.238(2.237–2.238)	<0.001	2.349(2.346–2.351)	<0.001
1927–1931	1.788(1.786–1.789)	<0.001	1.951(1.95–1.952)	<0.001	2.057(2.055–2.059)	<0.001
1932–1936	1.524(1.523–1.525)	<0.001	1.67(1.669–1.671)	<0.001	1.731(1.729–1.732)	<0.001
1937–1941	1.312(1.311–1.313)	<0.001	1.43(1.429–1.43)	<0.001	1.45(1.449–1.452)	<0.001
1942–1946	1.113(1.112–1.114)	<0.001	1.211(1.211–1.212)	<0.001	1.198(1.196–1.2)	<0.001
1947–1951	0.945(0.944–0.945)	<0.001	1.028(1.028–1.029)	<0.001	0.979(0.978–0.98)	<0.001
1952–1956	0.83(0.829–0.831)	<0.001	0.893(0.893–0.893)	<0.001	0.851(0.85–0.852)	<0.001
1957–1961	0.739(0.738–0.739)	<0.001	0.776(0.776–0.776)	<0.001	0.738(0.737–0.739)	<0.001
1962–1966	0.659(0.658–0.659)	<0.001	0.672(0.672–0.672)	<0.001	0.637(0.636–0.639)	<0.001
1967–1971	0.583(0.582–0.583)	<0.001	0.581(0.58–0.581)	<0.001	0.527(0.526–0.528)	<0.001
1972–1976	0.526(0.526–0.527)	<0.001	0.505(0.505–0.506)	<0.001	0.462(0.46–0.463)	<0.001
1977–1981	0.474(0.473–0.474)	<0.001	0.438(0.438–0.439)	<0.001	0.416(0.415–0.417)	<0.001
1982–1986	0.421(0.421–0.422)	<0.001	0.378(0.378–0.379)	<0.001	0.366(0.365–0.368)	<0.001
1987–1991	0.375(0.374–0.376)	<0.001	0.329(0.329–0.329)	<0.001	0.316(0.315–0.317)	<0.001
1992–1996	0.327(0.326–0.328)	<0.001	0.281(0.281–0.282)	<0.001	0.274(0.272–0.275)	<0.001
1997–2001	0.285(0.284–0.286)	<0.001	0.241(0.24–0.241)	<0.001	0.24(0.238–0.242)	<0.001
2002–2006	0.251(0.249–0.254)	<0.001	0.205(0.204–0.206)	<0.001	0.203(0.2–0.206)	<0.001

RRs, relative risk; IHD, Ischemic heart disease; CI, confidence interval.

### 3.9 Predictive analysis of the IHD

We used three methods to make cohort projections of ischemic heart disease epidemiology over the next 25 years (2021–2046). Overall, by middle 21st century, although the fluctuation changes predicted by the three methods are different, the number of people with the disease still has an upward trend. As for the standardization rate, the annual standard rate predicted by the three methods is generally quite different. The results of the three methods are described in detail below.

#### 3.9.1 BAPC analysis

**Figure 9:**
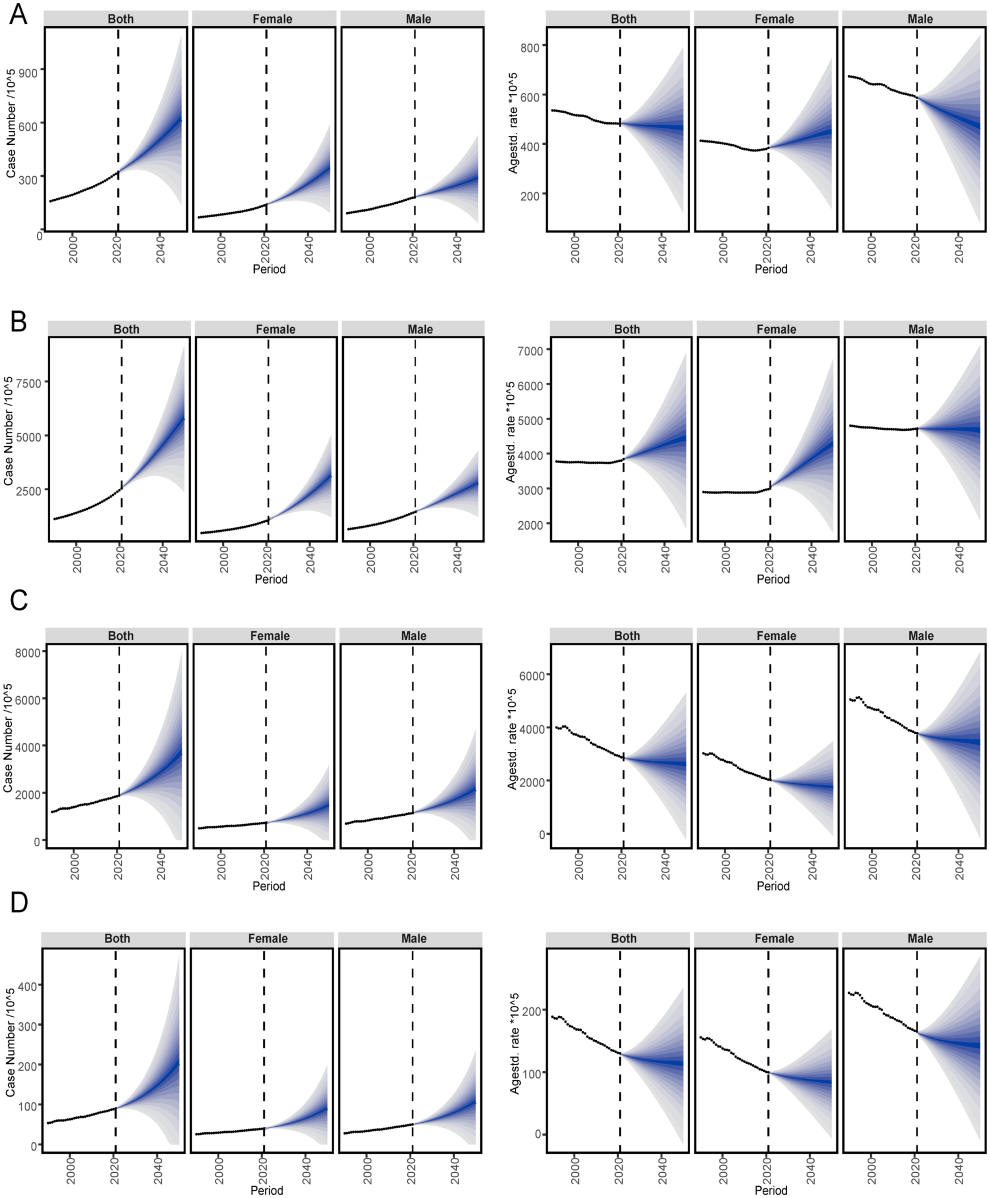
Global trends of IHD in ASR of incidence **(A)**, prevalence **(B)**, deaths **(C)**, and DALYs **(D)** rates from 2021 to 2046 by sex predicted by Arima models. DALYs, Disability-adjusted life years; GBD, Global Burden of Disease; UIs, Uncertainty interval.

The analysis of ischemic heart disease patients predicted to 2046 is shown in the figure. Globally, the case number of incidence of IHD increases slightly with each year, and by 2046 the number of patients is expected to reach 56,431,619 (both, 1.77 times of the number of cases in 2021), 30,294,495 (female, 2.18 times of the number of cases in 2021) and 26,264,574 (male, 1.46 times of the number of cases in 2021). The ASIR of IHD in groups both and male have a downward trend with the years, reaching about 459 per 100,000 population (1.23 times of the ASR in 2021), 476 per 100,000 population, respectively, by 2046 (1.57 times of the ASR in 2021), while the female group has a slight upward trend, reaching about 435 per 100,000 population (0.97 times of the ASR in 2021). The case number of prevalence of IHD increased slightly with the increase of years, and the number of patients is expected to reach 525,590,673 in both (2.07 times of the number of cases in 2021), 275,042,876 in female (2.52 times of the number of cases in 2021) and about 257,865,564 in male (1.77 times of the number of cases in 2021). The ASPR of IHD in groups both and female have a rising trend with the years, reaching 4,325 per 100,000 population (1.47 times of the ASR in 2021), 4,067 per 100,000 population, respectively, by 2046 (1.72 times of the ASR in 2021), while the male group has a steady and slightly decreasing trend, reaching 4,631 per 100,000 population (1.28 times of the ASR in 2021). The number of DALYs of IHD increased slightly with the increase of years, and by 2046, the the number of patients is estimated to reach about 331,439,474 (both, 1.76 times of the number of cases in 2021), 128,863,176 (female, 1.76 times of the number of cases in 2021) and 191,308,407 (male, 1.66 times of the number of cases in 2021). The ASDR of IHD decreased with each year, and estimated to reach about 2,576 per 100,000 population (both, 1.16 times of the ASR in 2021), 1742 per 100,000 population (female, 1.09 times of the ASR in 2021), and 3,383 per 100,000 population (male, 1.17 times of the ASR in 2021). The case number of deaths of IHD increases slightly with each year, and by 2046 the number of patients is expected to reach 17,541,188 (both, 1.95 times of the number of cases in 2021), 7,777,152 (female, 1.95 times of the number of cases in 2021) and 9,292,323 (male, 1.86 times of the ASR in 2021). The ASMR of IHD decreased with each year, and estimated to reach about 112 per 100,000 population (both,1.03 times of the ASR in 2021), 83 per 100,000 population (female, 0.97 times of the ASR in 2021), and 140 per 100,000 population (male, 1.02 times of the ASR in 2021; Figure 9; Supplementary Table S5).

#### 3.9.2 Nordpred analysis

**Figure 10:**
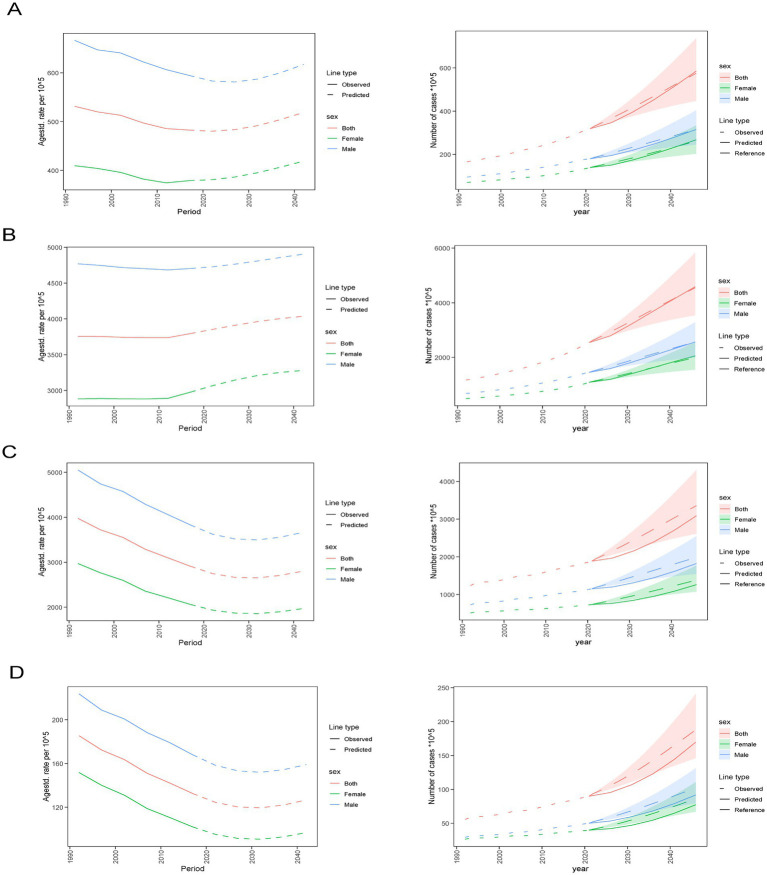
Global trends of IHD in case number and ASR of incidence **(A)**, prevalence **(B)**, deaths **(C)**, and DALYs **(D)** rates from 2021 to 2046 by sex predicted by Nordpred models. DALYs, Disability-adjusted life years, GBD, Global Burden of Disease, UIs, Uncertainty interval.

Globally, the case number of incidence, prevalence, mortality, and DALYs of IHDs have all increased over the years, with male patients outnumbering female patients, as shown in the figure. Overall, the ASR of incidence, prevalence, mortality, and DALYs for IHD have declined from 1990 to 2021. The predicted ASR of incidence and prevalence increased with the change in 2020. The predicted ASR trend of DALYs for IHD will continue to rise after 2030 (Figure 10; Supplementary Table S6).

#### 3.9.3 Arima analysis

**Figure 11:**
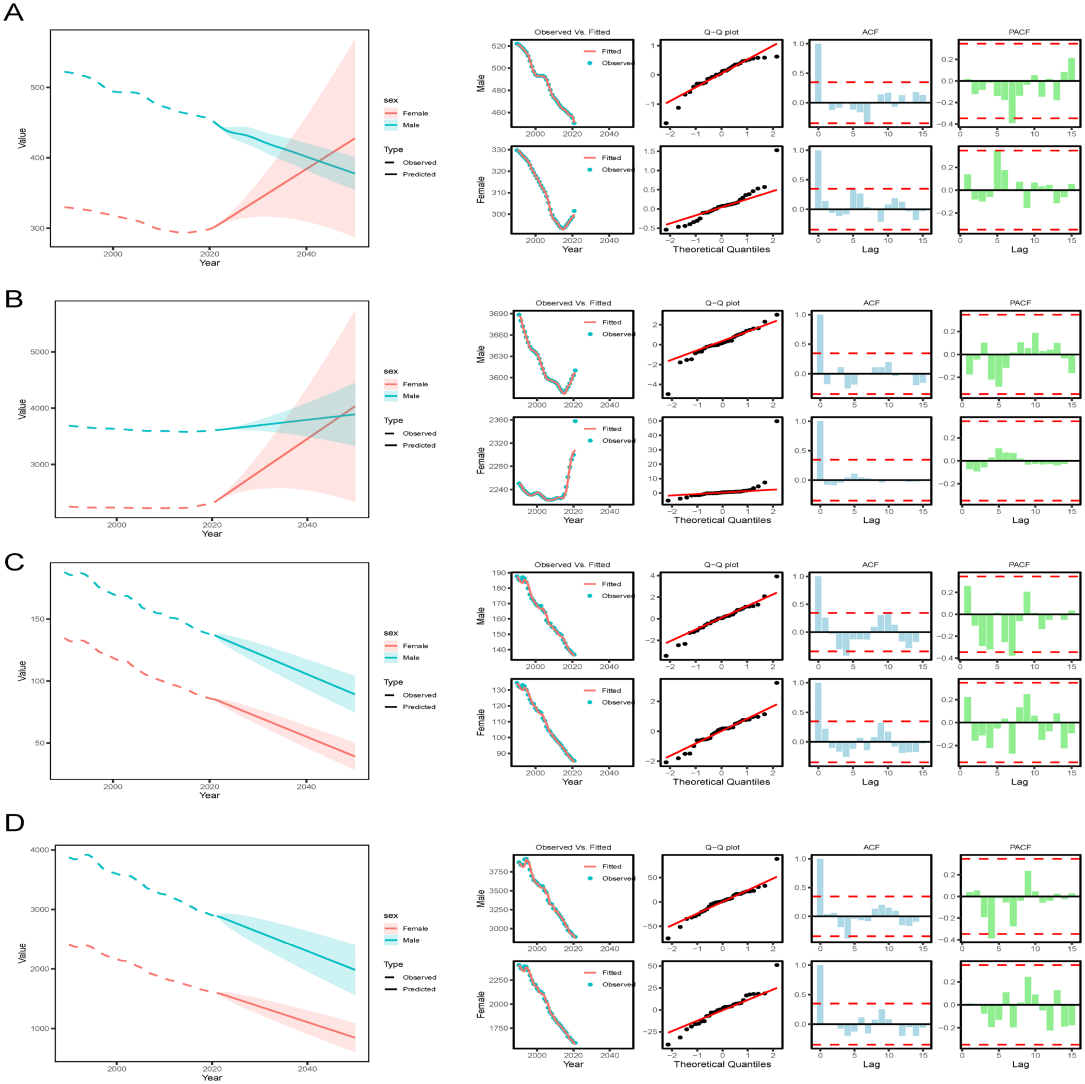
Global trends of IHD in case number and ASR of incidence **(A)**, prevalence **(B)**, deaths **(C)**, and DALYs **(D)** rates from 2021 to 2046 by sex predicted by Arima models. DALYs, Disability-adjusted life years, GBD, Global Burden of Disease, UIs, Uncertainty interval.

We used the Arima method to predict the ASR of incidence, prevalence, death, and DALYs of IHD in male and female groups. All four prediction models were consistent with the Arima detection, as shown in the figure for details. 2021, with the increase of years, the predicted ASIR value of men will decline after 2021, while that of women will increase, and the standard annual rate of women will exceed that of men by 2046. In terms of ASPR, both genders have seen an increase, and by 2046, the predicted value for females will be higher than that for males. As the years progress, the ASRs for both deaths and DALYs for both genders have decreased. By 2046, the value for males will be greater than that for females, according to the curve’s results (Figure 11; Supplementary Table S7).

## 4 Discussion

This study provides the most recent data on incidence, prevalence, mortality, and DALYs of IHD at global, regional, and country levels from 1990 to 2021 and further utilizes trend analysis, risk factors analysis, frontier analysis, analysis of health inequalities, and predictive analysis to process the data comprehensively. Similar to previous studies, we found that global ASR in Incidence and DALYs of IHD decreased in 2021 compared to 30 years ago and that prevalence and mortality increased slightly. Still, the overall case numbers continued to grow (30). Globally, in 2021, there were 31,872,778 cases of incidence of IHD (201.6%), 254,276,268 cases of prevalence of IHD (226.7%), 18,8,360,557 cases of DALYS of IHD (53.46%), and 5,367,137 deaths of IHD (40.3%) compared to 1990. From the global map of IHD, geographical and economic factors play a crucial role in the distribution and variation of IHD. For example, the incidence is high in Central Asia, East Asia, and Eastern Europe and low in Australia, Central Europe, high-income North America, and Western Europe. Vast geographic differences may be related to various factors, including race, socio-environmental exposure, urbanization, and dietary patterns (31–34). These findings can reference future intervention targets in different countries and regions.

The decomposition analysis shows that the increase in IHD is driven by population growth and aging, both globally and in most regions divided by SDI. In contrast, in high and high-middle SDI regions, it is mainly mediated by aging factors. Due to low fertility and higher levels of quality of care, the increasing older population and longer life expectancy in high, medium, and high SDI regions lead to an increased likelihood of chronic diseases such as ischemic heart disease, which creates more healthcare costs and human resources (35). Population growth has a significant effect on patients with ischemic heart disease in low SDI areas. As the population increases, these areas face challenges such as inadequate health care resources, unhealthy lifestyles, and increased social and family economic pressures, which increase the risk of IHD. Financial globalization has contributed to a further increase in the incidence of ischemic heart disease in these countries, primarily as a result of the adoption of Western dietary concepts and unhealthy lifestyle habits (36). Consequently, it is essential to enhance medical facilities, public health education, and living conditions in these areas to address the health challenges posed by population growth.

Furthermore, the decomposition results indicate that the three primary factors have a greater impact on male patients than female patients across all regions. This disparity may be attributed to higher rates of smoking and alcohol consumption among men, as well as a relatively low emphasis on health screening and adherence to medical recommendations. This requires healthcare professionals to conduct health education based on male and female patients’ different needs and risk factors, implement differentiated management, promote healthy eating, and encourage family members to participate to improve citizens’ heart health. With economic growth and improved living standards, dietary patterns in high-SDI and high-middle SDI regions have shifted from traditional healthy diets to Western fast food, characterized by higher calories, fat, and cholesterol, and processed foods with low nutritional value. This transition has resulted in increased cholesterol intake, contributing to a higher incidence of cardiovascular diseases. High-fat, high-cholesterol diets have been shown to exacerbate lipid disorders, promote the development and progression of atherosclerotic diseases, and lead to the occurrence of adverse events such as ischemic heart disease (37, 38). They are also one of the dietary patterns used in animal models of atherosclerotic disease (39). In addition, in high SDI and middle SDI areas, the rate of hypercholesterolemia in the older population is generally higher, and the metabolism of the older population decreases the digestion and absorption of the high-fat and high-cholesterol diet, increasing the risk of heart disease. Dietary habits of most Western countries have accelerated the rise in the population of ischemic heart disease, setting measures to address this issue. However, interestingly, our observation of the IHD incidence and EAPC map shows that Mediterranean people in Mediterranean countries, such as Slovenia, Cyprus, Spain, France and Greece, have endured relatively less pressure from illness, diet promoted by these countries has been supported by several clinical studies (40–42), including the famous Seven Countries Study by Keys (43), which has shown a reduction in the risk of various events such as ischemic heart disease, myocardial infarction, and stroke. This may be related to lower blood pressure and lipid levels, reduced oxidative stress, endothelial dysfunction, and vascular inflammation, thereby alleviating the degree of atherosclerosis (44, 45). In formulating dietary intervention programs, government agencies should collaborate with experts across diverse fields, including public health, nutrition, and sociology. They must closely monitor changes in dietary patterns in countries within the region, such as increasing the intake of fruits, vegetables, fish, seafood, legumes, and nuts, promoting olive oil in cooking, and encouraging the consumption of antioxidant-rich foods. At the same time, effective intervention strategies should be implemented to reduce the risk of diseases caused by suboptimal dietary habits. In the low-middle SDI regions, particulate matter pollution has become a leading factor contributing to IHD deaths. Research indicates that long-term exposure to particulate matter is significantly associated with an increased incidence of various diseases, including IHD. Particulate matter can induce the accumulation of monocytes in atherosclerotic plaques, trigger oxidative stress cascades, accelerate the early stages of atherosclerosis, and promote coronary artery calcification. These processes increase the risk of heart disease and are strongly associated with mortality and disability from IHD in males (46–48). Energy organizations should enhance air quality monitoring, promote clean energy initiatives, improve public transportation systems, advocate for sustainable urban planning, conduct health education campaigns, foster cross-sectoral collaboration, reduce particulate pollution, and ultimately improve health outcomes for individuals in low SDI regions (49). Regarding gender, women in high-income and high-middle SDI areas exhibit greater susceptibility to blood pressure-related risks associated with environmental occupations. This increased vulnerability may stem from biological differences and familial and occupational pressures (50).

As the global economy expands, disparities in development levels among countries and regions continue to widen. Workers in low SDI areas are predominantly involved in high-risk manual labor occupations, such as mining, construction, and manufacturing. These occupational environments frequently have harmful chemicals, noise, and other health hazards. Prolonged exposure to such factors may elevate the risk of cardiovascular disease (51–53). Furthermore, the healthcare infrastructure in low SDI areas is relatively inadequate. The absence of effective disease prevention and treatment measures hampers the population’s access to timely and appropriate medical services, thereby increasing the disease risk. In the risk factor analysis for 2021, we also found that renal dysfunction had entered the list of important risk factors for disability from ischemic heart disease. Impaired kidney function can disrupt the clotting system, forming thrombosis precursors and thereby increasing the risk of ischemic events (54). The inflammatory process associated with CKD can also lead to endothelial dysfunction and atherosclerosis, increasing the IHD risk (55).

During the period from 1990 to 1994, the increase in mortality and disability rates was primarily due to inadequate medical conditions, insufficient rescue capacity, and a lack of understanding of diseases. Many patients were already in a severe stage of illness by the time of diagnosis, and the lack of effective treatment and rehabilitation measures led to high mortality and disability rates. Between 2005 and 2014, the incidence of cardiovascular diseases increased, which can be attributed to urbanization and economic development. This trend reflects the pressure and adverse effects of economic growth and lifestyle changes on public health. The incidence of cardiovascular diseases significantly increased once again during the 2015–2019 period, primarily due to unhealthy modern lifestyles, such as sedentary behavior, elevated stress levels, and poor dietary habits, along with accelerated aging, which exposed a larger population of older adults to the risk of heart disease (56, 57). In short, the trend of ischemic heart disease reflects the impact of factors such as social and economic development, medical technology advancement, lifestyle changes, and aging. It can be seen that comprehensive, preventive, and community engagement strategies are essential to control and prevent ischemic heart disease. The results of the age-period cohort suggest that the prevalence risk of IHD peaks among older men aged 75–79 and that the incidence and death rates increase significantly with age. Between 1990 and 2021, the risk of IHD has demonstrated a persistent upward trend, particularly highlighting a significant increase in recent years. This trend may be associated with the COVID-19 pandemic and its complications (58, 59). Simultaneously, as the birth cohort progresses, the risk of IHD declines, suggesting that advancements in medical treatment and improvements in lifestyle have had a positive effect. These findings emphasize the importance of conducting year, time, and cohort studies in formulating comprehensive prevention strategies for ischemic heart disease.

Over the next 25 years, while the changes in the standardized annual rates of four indicators predicted by the three methods vary, the overall number of cases is expected to rise. Globally, the incidence of ischemic heart disease is projected to increase slightly over time, with a higher prevalence in men compared to women. However, the future trend of women in multiple indicators shows an upward trend, warranting attention. This trend may be associated with cardiovascular physiological changes in women following menopause, necessitating a focus on appropriate clinical measures (60, 61). Furthermore, the discrepancies in the predictions of various indicators by the three trend forecasting analyses highlight the diversity and complexity of future trends in IHD. Health departments of all countries should proactively enhance the core capabilities of primary health systems, including risk screening, drug supply, and chronic disease management, and make differentiated investments in human resources and facilities based on the regional development levels. When formulating policies, governments should focus on key populations and risk factors and customize health management paths for postmenopausal women. In high and medium-development regions, efforts should be made to deepen healthy diet, tobacco control, and metabolic management. In contrast, in low and medium and low development regions, air quality improvement, occupational safety assurance, and kidney health management should be placed at the core of prevention and control. Given the complexity of the prediction results, government agencies of all countries should fully utilize artificial intelligence technology to establish, optimize, and promote epidemiological prediction models for chronic diseases such as ischemic heart disease, promote cross-border experience exchanges in areas such as acute event response and secondary prevention, and systematically evaluate the cost-effectiveness of various intervention measures, to jointly address this global health challenge (62, 63).

Compared with the relatively single methods of previous GBD studies (such as GBD 2017/2019), which overly relied on descriptive statistical analysis, this study is based on the latest data from GBD 2021. It employs a variety of analytical tools and predictive models to conduct a comprehensive and in-depth analysis of the burden of ischemic heart disease (IHD). At the same time, meticulous risk factor assessment reveals emerging risk factors such as kidney dysfunction and low-grain diet and tailors precise intervention strategies for different social regions. This research lays a solid empirical foundation for the dynamic risk prediction of global ischemic heart disease and provides valuable scientific evidence for regionalized precise intervention. However, there are some limitations in the GBD study. Although the GBD database encompasses multiple data sources globally, the quality and completeness of data may vary across different countries and regions, which could impact the accuracy and reliability of the results. Despite the robust statistical methods employed in the study to mitigate these effects, issues such as inadequate registration of ischemic heart disease, data omissions, and scarcity of medical resources in some underdeveloped countries in Africa and Asia still lead to a certain degree of data bias. In addition, the conclusions drawn from the GBD original data using some statistical methods such as Joinpoint analysis and the viewpoints generated based on previous literature need to be interpreted carefully in combination with current research literature, social background, policies, etc. In the future, High-income countries, emerging economies, low-income countries, and some African countries show different patterns in terms of IHD burden, which deserves a more in-depth stratified analysis. In addition, our team will further collaborate with other databases to conduct multiple verifications to ensure the reliability of the results.

## 5 Conclusion

We emphasize that, in formulating health policies and comprehensively understanding the global and regional burden of IHD, it is essential to prioritize risk factors in high-risk areas to identify vulnerable populations, address age- and sex-specific disparities, and predict disease trends. These findings should directly inform health policy development, for instance: (1) Regional risk factor prioritization should guide future health policies to enable targeted resource allocation for interventions; (2) Tailored prevention strategies should be implemented for high-risk groups (specific age or sex cohorts) to enhance intervention efficiency. Such measures will help mitigate IHD progression, reduce healthcare burdens, and provide evidence-based support for optimizing medical resource distribution, clinical decision-making, and public health policy formulation.

## Data Availability

The original contributions presented in the study are included in the article/Supplementary material, further inquiries can be directed to the corresponding author.

## Footnotes

^1^ http://ghdx.healthdata.org/gbd-results-tool
