# Supplementary material for: Trends and predictive research on the global burden of ischemic heart disease from 1990 to 2021: an analysis of the Global Burden of Disease study 2021
Source: Front Public Health. 2025 Sep 19;13:1569179. doi: 10.3389/fpubh.2025.1569179 (PMC12491020; doi:10.3389/fpubh.2025.1569179)
Supplement: Supplementary file 1 [file Supplementary_file_1.docx]

**Supplementary methods**

We conduct literature search for the detailed content of other methods such as decomposition analysis, and take DALYs as an example to explain the detailed method.

**Decomposition analysis:**

We first used the decomposition methodology of Das Gupta^[1-3](#_ENREF_1)^ to decompose IHD DALYs by population age structure, population growth, and epidemiologic changes. The number of DALYs at each location was obtained from the following formula:

DALY _ay, py, ey_ = $\sum_{i=1}^{20} ($a _i, y_ * p _y_ * e _i, y_)

Where DALY _ay, py, ey_ represented DALYs based on the factors of age structure, population, and DALYs rate for specific year y; a _i_ _y_ represents the proportion of population for the age category i of the 20 age categories in given year y; p _y_ represents the total population in given year y; and e _i, y_ represents DALYs rate given age category i in year y. The contribution of each factor to the change in DALYs from 1990 to 2021 was defined by the effect of one factor changing while the other factors were held constant. For example, the effect of age structure was calculated as:

[(DALY _a2021, p1990, e1990_ + DALY _a2021, p2021, e2021_)/3+ (DALY _a2021, p1990, e2021_ + DALY _a2021, p2021, e1990_)/6] - [(DALY _a1990, p2021, e2021_ + DALY _a1990, p1990, e1990_)/3+ (DALY _a1990, p2021b, e1990_ + DALY _a1990, p1990, e2021_)/6]

**Frontier Analysis:**

In order to evaluate the relationship between burden of IHD and socio-demographic development, we applied a frontier analysis as a quantitative methodology to identify the lowest potentially achievable age-standardized DALYs rate on the basis of development status as measured by the Socio-demographic Index (SDI). The DALYs frontier pinpoints the minimum DALYs that could be attained for every country or territory given its SDI. Distance from the frontier is termed effective difference; a large effective difference from the frontier suggests there may be unrealized opportunities for gains or improvement (reduction in IHD DALYs) that should be possible based on the country or territory’s place on the development spectrum. A data envelope analysis, which allows for the delineation of non-linear frontiers, utilizing the free disposal hull method was developed to produce a frontier for age-adjusted IHD DALYs by SDI^4,5^ using data from 1990-2021. In order to account for uncertainty, we used 1,000 bootstrapped samples of the data, randomly sampling with replacement from all countries and territories in all years. Mean IHD DALYs at each SDI value from the bootstrapped samples was computed. LOESS regression with local polynomial degree of 1 and span of 0.2 was then developed to generate a smoothed frontier^[4](#_ENREF_4)^. To exclude influence of outliers, super-efficient countries were excluded in the generation of the frontier^[4](#_ENREF_4)^. To understand the relationship of age-standardized IHD DALYs rates vis-à-vis the frontier in 2021, we calculated the effective difference (the absolute distance from the frontier) using 2021 SDI and age-standardized IHD DALYs rate data point for each country or territory. Countries or territories with lower DALYs than the frontiers were assigned a zero distance.

**References:**

S1. P. DG. *Standardization and decomposition of rates: a user’s manual, Pages 19-36*, 1993.

S2. Das Gupta P. Standardization and decomposition of rates from cross-classified data. *Genus* 1994; **50:** 171-196.

S3. Chevan A, Sutherland M. Revisiting Das Gupta: refinement and extension of standardization and decomposition. *Demography* 2009; **46:** 429-449.

S4. Access GBDH, Quality Collaborators. Electronic address cue, Access GBDH*, et al.* Healthcare Access and Quality Index based on mortality from causes amenable to personal health care in 195 countries and territories, 1990-2015: a novel analysis from the Global Burden of Disease Study 2015. *Lancet* 2017.

S5. Xie Y, Bowe B, Xian H*, et al.* Rate of Kidney Function Decline and Risk of Hospitalizations in Stage 3A IHD. *Clinical journal of the American Society of Nephrology : CJASN* 2015; **10:** 1946-1955.
