# Supplementary material for: Trends and predictive research on the global burden of ischemic heart disease from 1990 to 2021: an analysis of the Global Burden of Disease study 2021
Source: Front Public Health. 2025 Sep 19;13:1569179. doi: 10.3389/fpubh.2025.1569179 (PMC12491020; doi:10.3389/fpubh.2025.1569179)
Supplement: Supplementary file 9 [file Table_8.docx]

| **location** | **1990** | | **2021** | | EAPC(95%UI) 1990-2021 |
| --- | --- | --- | --- | --- | --- |
|  | Number (95% UI) | ASR (95% UI) | Number (95% UI) | ASR (95% UI) |  |
| Global | 15813618.648 (13180529.438,18849478.54) | 419.539 (351.067,498.146) | 31872778.179 (26284920.941,38267834.295) | 372.901 (307.95,444.193) | -0.441 (-0.468,-0.414) |
| **SDI quintiles** |  |  |  |  |  |
| High SDI | 3768111.277 (3189322.459,4475353.957) | 343.51 (290.807,407.034) | 3989835.191 (3384023.956,4709126.259) | 195.63 (164.52,231.528) | -2.044 (-2.288,-1.8) |
| High-middle SDI | 4242517.6 (3542999.273,5036433.887) | 462.739 (386.71,546.341) | 7874112.387 (6457244.149,9448767.955) | 404.438 (331.924,480.976) | -0.58 (-0.692,-0.466) |
| Middle SDI | 3654917.793 (2965633.955,4446596.133) | 382.771 (314.262,459.865) | 10499399.857 (8567480.072,12702596.067) | 403.841 (330.368,481.688) | 0.218 (0.15,0.286) |
| Low SDI | 1004366.901 (814679.074,1236950.522) | 471.405 (384.069,572.6) | 2190922.834 (1807960.132,2673792.332) | 444.607 (362.902,537.75) | -0.304 (-0.365,-0.243) |
| Low-middle SDI | 3125084.87 (2574308.177,3805366.475) | 531.544 (440.443,637.687) | 7292860.054 (6121118.776,8702139.94) | 515.602 (433.638,614.953) | -0.091 (-0.134,-0.048) |
| **GBD regions** |  |  |  |  |  |
| Andean Latin America | 50619.461 (41422.009,61339.63) | 249.989 (203.016,303.512) | 139102.859 (113541.583,169610.001) | 233.086 (188.744,282.95) | -0.308 (-0.386,-0.229) |
| Australasia | 84214.365 (73573.84,97267.832) | 365.936 (321.529,421.768) | 116017.25 (95474.519,139639.292) | 220.627 (181.556,267.7) | -1.7 (-1.996,-1.403) |
| Caribbean | 104244.177 (87044.169,122816.663) | 412.475 (346.607,484.795) | 195155.804 (161425.864,231734.544) | 360.967 (298.085,428.102) | -0.481 (-0.594,-0.368) |
| Central Asia | 281995.468 (245768.846,329015.893) | 641.968 (560.798,735.591) | 573033.86 (510496.846,653368.009) | 801.563 (731.973,893.803) | 0.7 (0.538,0.862) |
| Central Europe | 697968.125 (609103.333,805202.082) | 494.653 (434.027,565.427) | 705225.411 (612792.64,813235.108) | 322.439 (280.414,371.499) | -1.817 (-2.037,-1.596) |
| Central Latin America | 280079.587 (229936.403,338385.41) | 343.143 (279.831,415.187) | 775551.033 (634023.615,938828.82) | 309.821 (253.976,372.648) | -0.423 (-0.487,-0.36) |
| Central Sub-Saharan Africa | 77096.591 (63116.052,94967.978) | 373.704 (310.544,445.964) | 181545.34 (148129.003,222440.836) | 344.019 (286.709,408.906) | -0.388 (-0.429,-0.347) |
| East Asia | 2400602.995 (1941066.764,2911498.834) | 316.288 (256.633,382.833) | 7541711.463 (6010952.737,9229492.799) | 363.645 (291.859,437.509) | 0.621 (0.467,0.774) |
| Eastern Europe | 1835281.036 (1497315.733,2193024.821) | 710.561 (581.309,851.384) | 2491976.745 (2009144.616,3043098.956) | 714.219 (578.979,859.744) | -0.226 (-0.497,0.045) |
| Eastern Sub-Saharan Africa | 224084.498 (177674,279662.755) | 322.836 (254.709,398.76) | 516095.99 (414727.128,642412.662) | 314.401 (250.546,386.714) | -0.217 (-0.272,-0.161) |
| High-income Asia Pacific | 206093.994 (166290.483,251344.124) | 106.591 (86.549,129.282) | 460224.262 (366622.258,577587.816) | 92.403 (73.463,115.102) | -0.581 (-0.786,-0.375) |
| High-income North America | 1581463.531 (1246575.069,1975540.915) | 454.955 (360.35,569.191) | 1114553.157 (941548.833,1307276.563) | 174.123 (147.073,203.067) | -3.548 (-3.832,-3.263) |
| North Africa and Middle East | 1595178.367 (1382346.646,1887102.916) | 984.292 (859.82,1153.004) | 4046958.298 (3504191.994,4785078.672) | 895.847 (786.655,1043.487) | -0.415 (-0.491,-0.339) |
| Oceania | 9458.731 (7342.633,11898.012) | 375.427 (291.966,470.915) | 25258.354 (20071.869,31437.982) | 378.318 (297.304,469.959) | 0.035 (0.019,0.05) |
| South Asia | 3343753.625 (2669988.976,4119600.508) | 600.214 (482.647,736.668) | 8437391.687 (6853498.849,10223098.748) | 580.236 (472.942,704.587) | -0.129 (-0.213,-0.044) |
| Southeast Asia | 551918.438 (446571.922,668746.903) | 239.913 (198.424,287.224) | 1419034.864 (1164938.495,1720664.745) | 231.582 (193.091,275.946) | -0.052 (-0.127,0.022) |
| Southern Latin America | 139690.409 (120951.913,162646.166) | 315.009 (275.925,362.587) | 174479.231 (150037.872,203601.784) | 201.477 (172.901,236.15) | -1.681 (-1.942,-1.419) |
| Southern Sub-Saharan Africa | 106341.411 (84122.914,132913.219) | 403.208 (317.746,504.391) | 213190.343 (168672.024,266327.124) | 378.485 (299.198,467.871) | -0.362 (-0.441,-0.283) |
| Tropical Latin America | 180542.097 (151059.063,214652.648) | 196.85 (162.954,234.665) | 431283.868 (349771.473,518457.947) | 167.802 (136.408,200.758) | -0.366 (-0.443,-0.289) |
| Western Europe | 1753090.793 (1575512.261,1973071.483) | 305.364 (274.348,343.385) | 1596981.992 (1383367.481,1853127.359) | 172.456 (147.8,203.442) | -1.894 (-2.004,-1.784) |
| Western Sub-Saharan Africa | 309900.947 (247585.971,383976.467) | 376.076 (296.956,463.504) | 718006.368 (581487.342,884861.591) | 379.249 (303.356,464.666) | -0.065 (-0.104,-0.027) |
