# Supplementary material for: Trends and predictive research on the global burden of ischemic heart disease from 1990 to 2021: an analysis of the Global Burden of Disease study 2021
Source: Front Public Health. 2025 Sep 19;13:1569179. doi: 10.3389/fpubh.2025.1569179 (PMC12491020; doi:10.3389/fpubh.2025.1569179)
Supplement: Supplementary file 10 [file Table_9.docx]

| **location** | **1990** | | **2021** | | EAPC(95%UI) 1990-2021 |
| --- | --- | --- | --- | --- | --- |
|  | Number (95% UI) | ASR (95% UI) | Number (95% UI) | ASR (95% UI) |  |
| Global | 112169488.435 (99416740.976,125730168.733) | 2904.718 (2575.986,3248.097) | 254276267.852 (221446458.085,295493092.658) | 2946.379 (2572.688,3424.318) | 0.001 (-0.026,0.028) |
| **SDI quintiles** |  |  |  |  |  |
| High SDI | 25246967.805 (22384997.7,28275596.816) | 2271.859 (2020.891,2539) | 34231046.168 (30152992.709,39201712.323) | 1671.604 (1475.876,1910.426) | -1.212 (-1.357,-1.066) |
| High-middle SDI | 30745716.463 (27332573.295,34434266.971) | 3197.562 (2857.642,3581.489) | 63826218.254 (55672804.808,74555301.048) | 3217.576 (2814.342,3742.18) | -0.056 (-0.095,-0.017) |
| Middle SDI | 27913691.163 (24588240.351,31686021.27) | 2868.41 (2526.851,3236.767) | 85371063.071 (73614482.54,100118565.059) | 3226.303 (2806.668,3798.241) | 0.394 (0.365,0.423) |
| Low SDI | 6620304.681 (5896789.468,7438261.238) | 3100.354 (2764.327,3477.557) | 15420053.335 (13308339.222,17818345.522) | 3162.535 (2763.302,3679.596) | 0.027 (0.015,0.039) |
| Low-middle SDI | 21499369.593 (19030104.647,24197902.242) | 3717.205 (3292.295,4187.058) | 55198197.201 (48035746.014,63649428.134) | 3941.422 (3448.943,4577.028) | 0.215 (0.197,0.233) |
| **GBD regions** |  |  |  |  |  |
| Andean Latin America | 411051.435 (374685.351,453409.078) | 2041.829 (1855.495,2255.333) | 1320142.306 (1175584.066,1473053.76) | 2236.344 (1992.493,2499.741) | 0.342 (0.287,0.397) |
| Australasia | 575088.437 (531489.946,625054.097) | 2451.649 (2266.935,2660.322) | 1055922.112 (937657.929,1198106.748) | 1966.987 (1749.656,2225.895) | -0.847 (-1.014,-0.679) |
| Caribbean | 817912.231 (748442.722,895355.172) | 3182.403 (2910.397,3484.142) | 1719015.239 (1545788.039,1913703.127) | 3183.999 (2861.959,3542.79) | -0.038 (-0.104,0.027) |
| Central Asia | 1826093.86 (1697743.416,1985355.884) | 4095.928 (3802.281,4441.373) | 3360424.57 (3054199.108,3688626.419) | 4408.384 (4040.592,4801.108) | 0.237 (0.207,0.267) |
| Central Europe | 5510994.857 (4914268.31,6121939.005) | 3763.214 (3383.239,4156.9) | 7088998.322 (6244109.9,7996466.61) | 3192.858 (2824.44,3571.805) | -0.766 (-0.873,-0.659) |
| Central Latin America | 2186085.618 (1964375.121,2431257.025) | 2694.287 (2423.736,2989.191) | 6550181.114 (5738031.516,7491879.675) | 2625.168 (2306.273,2997.161) | -0.146 (-0.184,-0.109) |
| Central Sub-Saharan Africa | 471511.644 (428138.548,519525.386) | 2274.814 (2061.014,2502.924) | 1121950.86 (1003281.573,1251660.219) | 2151.753 (1934.112,2416.456) | -0.24 (-0.277,-0.202) |
| East Asia | 20306815.53 (17497234.218,23369855.747) | 2534.098 (2206.042,2917.752) | 65378738.592 (55703355.192,78423916.879) | 3031.244 (2597.744,3606.161) | 0.614 (0.534,0.695) |
| Eastern Europe | 12233736.379 (10760238.418,13888310.894) | 4531.317 (3997.321,5112.987) | 17417637.441 (15088871.107,20450190.523) | 4942.65 (4299.113,5766.775) | 0.204 (0.118,0.29) |
| Eastern Sub-Saharan Africa | 1511536.306 (1341342.313,1689486.043) | 2114.081 (1870.696,2361.788) | 3667714.673 (3201109.299,4187513.083) | 2224.884 (1931.931,2554.41) | 0.116 (0.096,0.136) |
| High-income Asia Pacific | 1927509.987 (1676871.703,2219259.271) | 967.928 (844.871,1108.659) | 3826068.673 (3304656.668,4472351.473) | 821.725 (714.58,948.262) | -0.768 (-0.88,-0.657) |
| High-income North America | 10084084.584 (8571681.531,11762328.992) | 2850.669 (2436.072,3310.935) | 9762662.998 (8311763.029,11497495.756) | 1494.62 (1280.2,1746.897) | -2.467 (-2.656,-2.277) |
| North Africa and Middle East | 10286345.487 (9619257.939,11065821.849) | 6435.15 (6033.547,6897.616) | 28353618.488 (25902216.059,31330581.988) | 6404.837 (5872.02,7041.083) | -0.067 (-0.095,-0.04) |
| Oceania | 71569.396 (65254.732,78323.621) | 2810.978 (2578.697,3077.139) | 193152.316 (172499.752,215291.785) | 2912.093 (2623.816,3229.738) | 0.145 (0.124,0.165) |
| South Asia | 22654345.788 (19415380.396,26136299.226) | 4173.322 (3601.464,4808.33) | 64277536.038 (54216694.537,76364441.793) | 4455.734 (3796.727,5339.205) | 0.218 (0.209,0.226) |
| Southeast Asia | 4659271.922 (4164301.608,5206583.6) | 2001.925 (1796.896,2238.149) | 12934270.768 (11289451.11,14655743.13) | 2088.432 (1847.375,2376.728) | 0.166 (0.139,0.192) |
| Southern Latin America | 832055.74 (761051.961,919385.781) | 1805.872 (1654.581,1985.537) | 1342051.299 (1209575.717,1499258.083) | 1538.85 (1388.102,1718.574) | -0.709 (-0.798,-0.619) |
| Southern Sub-Saharan Africa | 743247.24 (646889.945,849314.827) | 2858.768 (2487.01,3285.625) | 1557029.018 (1326447.581,1829630.197) | 2780.691 (2387.17,3277.901) | -0.192 (-0.246,-0.137) |
| Tropical Latin America | 1768219.905 (1514249.373,2025632.505) | 1954.508 (1676.105,2253.783) | 5084851.691 (4282245.689,5980523.646) | 1977.988 (1672.378,2324.962) | 0.067 (0.037,0.097) |
| Western Europe | 11268545.905 (10086320.214,12564352.983) | 1947.719 (1747.808,2162.574) | 13285326.744 (11713332.85,15180897.238) | 1480.144 (1305.918,1679.624) | -1.047 (-1.113,-0.981) |
| Western Sub-Saharan Africa | 2023466.183 (1790034.584,2270457.918) | 2399.144 (2121.658,2703.684) | 4978974.591 (4339864.008,5706176.195) | 2624.72 (2284.476,3000.353) | 0.318 (0.299,0.337) |
