# Supplementary material for: Trends and predictive research on the global burden of ischemic heart disease from 1990 to 2021: an analysis of the Global Burden of Disease study 2021
Source: Front Public Health. 2025 Sep 19;13:1569179. doi: 10.3389/fpubh.2025.1569179 (PMC12491020; doi:10.3389/fpubh.2025.1569179)
Supplement: Supplementary file 11 [file Table_10.docx]

| **location** | **1990** | | **2021** | | EAPC(95%UI) 1990-2021 |
| --- | --- | --- | --- | --- | --- |
|  | Number (95% UI) | ASR (95% UI) | Number (95% UI) | ASR (95% UI) |  |
| Global | 119162957.3 (114547786.892,123454733.022) | 3107.606 (2966.496,3222.674) | 188360557.323 (177036930.413,198154476.565) | 2212.163 (2075.545,2327.613) | -1.203 (-1.254,-1.153) |
| **SDI quintiles** |  |  |  |  |  |
| High SDI | 32005742.159 (30325688.872,32877811.045) | 2919.497 (2762.746,3002.023) | 23546711.437 (21449543.862,24737628.799) | 1134.02 (1053.561,1186.528) | -3.257 (-3.378,-3.136) |
| High-middle SDI | 33569030.727 (32291953.73,34708784.594) | 3589.764 (3435.607,3712.834) | 44542561.459 (41134046.45,48044417.492) | 2301.492 (2122.973,2482.464) | -1.74 (-2.007,-1.472) |
| Middle SDI | 26527074.227 (25117637.572,28067547.638) | 2593.455 (2447.246,2745.559) | 61242796.65 (56964813.149,65533800.611) | 2351.212 (2174.839,2514.627) | -0.274 (-0.324,-0.223) |
| Low SDI | 6194944.855 (5447647.401,6954569.822) | 2668.94 (2361.301,2967.214) | 12778296.891 (11572088.23,14121058.945) | 2464.12 (2235.866,2725.196) | -0.262 (-0.344,-0.18) |
| Low-middle SDI | 20676778.516 (19223601.748,22114207.224) | 3221.761 (2993.327,3437.467) | 46062373.822 (42687627.645,49488217.32) | 3138.581 (2912.652,3360.623) | 0.021 (-0.037,0.08) |
| **GBD regions** |  |  |  |  |  |
| Andean Latin America | 389626.651 (355358.538,430137.252) | 1858.091 (1696.986,2045.717) | 681780.759 (578423.087,811744.203) | 1150.606 (976.724,1369.618) | -1.766 (-2.156,-1.374) |
| Australasia | 752012.8 (712619.35,773987.972) | 3237.018 (3058.203,3338.897) | 446236.555 (400159.583,474158.622) | 812.686 (739.289,858.374) | -4.68 (-4.821,-4.538) |
| Caribbean | 950094.484 (906314.851,988524.899) | 3723.19 (3551.609,3873.174) | 1291386.799 (1138002.54,1470658.95) | 2397.798 (2112.339,2730.367) | -1.425 (-1.634,-1.216) |
| Central Asia | 2808714.981 (2697191.799,2899667.306) | 6206.854 (5937.366,6407.426) | 3674595.535 (3323875.255,4049458.434) | 4864.49 (4415.547,5338.748) | -1.312 (-1.66,-0.964) |
| Central Europe | 7442730.741 (7251102.091,7571719.755) | 5198.333 (5046.133,5293.629) | 5524245.141 (5103797.442,5888037.3) | 2471.231 (2288.198,2635.382) | -2.746 (-2.862,-2.631) |
| Central Latin America | 2013987.925 (1954877.935,2051366.587) | 2445.659 (2357.942,2497.278) | 5014766.53 (4523533.435,5572802.53) | 2016.965 (1821.529,2238.284) | -0.82 (-1.064,-0.576) |
| Central Sub-Saharan Africa | 610385.442 (476421.932,774810.188) | 2816.499 (2225.44,3535.982) | 1296119.192 (1001600.068,1666371.532) | 2433.064 (1902.528,3100.829) | -0.645 (-0.729,-0.56) |
| East Asia | 14164879.801 (12519625.698,16050196.244) | 1770.784 (1573.998,1979.397) | 36779980.991 (30982599.338,42822726.051) | 1839.923 (1541.205,2135.322) | 0.465 (0.205,0.726) |
| Eastern Europe | 15693814.332 (15174105.893,16002683.873) | 5957.771 (5735.977,6088.415) | 16349319.098 (14850165.179,17851788.069) | 4687.698 (4267.785,5115.681) | -1.34 (-1.886,-0.792) |
| Eastern Sub-Saharan Africa | 1209724.426 (1076473.776,1395912.915) | 1556.442 (1388.667,1773.022) | 2710701.18 (2345578.691,3122231.023) | 1536.641 (1333.116,1773.889) | -0.249 (-0.34,-0.156) |
| High-income Asia Pacific | 2221306.677 (2085563.916,2305288.636) | 1165.807 (1080.718,1212.459) | 2313089.597 (2010801.769,2492541.917) | 492.99 (446.933,520.219) | -2.754 (-2.84,-2.668) |
| High-income North America | 11711568.101 (10945809.4,12090045.186) | 3347.441 (3141.102,3449.541) | 9426914.825 (8621683.567,9884596.771) | 1461.897 (1350.513,1526.214) | -2.985 (-3.139,-2.83) |
| North Africa and Middle East | 9832454.932 (9159577.173,10677658.795) | 5763.466 (5366.025,6251.516) | 18148597.815 (16140157.225,20526102.821) | 4023.223 (3581.712,4507.47) | -1.237 (-1.276,-1.199) |
| Oceania | 136528.312 (111593.748,168663.781) | 4259.046 (3546.41,5124.643) | 327008.342 (270023.064,393473.359) | 3962.772 (3323.164,4723.707) | -0.195 (-0.247,-0.142) |
| South Asia | 20470723.866 (18722018.46,22243363.896) | 3276.846 (2983.931,3564.633) | 50666052.864 (46308000.427,54713621.318) | 3351.094 (3075.412,3616.422) | 0.165 (0.067,0.264) |
| Southeast Asia | 6837277.505 (6257622.455,7403583.107) | 2549.168 (2315.762,2761.717) | 15931224.368 (14296723.024,17482653.924) | 2415.55 (2177.873,2635.297) | -0.163 (-0.223,-0.104) |
| Southern Latin America | 1264775.091 (1223961.933,1297794.187) | 2821.47 (2718.033,2901.049) | 931016.804 (877897.191,968371.97) | 1070.856 (1012.572,1113.222) | -2.863 (-3.008,-2.717) |
| Southern Sub-Saharan Africa | 452076.822 (404055.32,494626.25) | 1626.436 (1424.737,1789.709) | 957913.546 (887404.652,1042420.522) | 1689.527 (1565.065,1832.244) | 0.107 (-0.316,0.533) |
| Tropical Latin America | 2675370.301 (2581470.811,2739000.566) | 2899.865 (2774.077,2979.355) | 3817675.011 (3591188.645,3962634.952) | 1476.136 (1385.484,1533.172) | -2.127 (-2.202,-2.053) |
| Western Europe | 15770965.177 (14972578.333,16197676.414) | 2740.647 (2604.658,2812.945) | 8262192.401 (7385900.607,8764788.263) | 843.767 (775.271,886.103) | -3.999 (-4.104,-3.894) |
| Western Sub-Saharan Africa | 1753938.933 (1491914.439,2043990.402) | 2082.33 (1785.443,2418.297) | 3809739.969 (3233241.86,4452024.134) | 2029.03 (1760.435,2334.059) | -0.1 (-0.245,0.045) |
