# Supplementary material for: Trends and predictive research on the global burden of ischemic heart disease from 1990 to 2021: an analysis of the Global Burden of Disease study 2021
Source: Front Public Health. 2025 Sep 19;13:1569179. doi: 10.3389/fpubh.2025.1569179 (PMC12491020; doi:10.3389/fpubh.2025.1569179)
Supplement: Supplementary file 12 [file Table_11.docx]

| **location** | **1990** | | **2021** | | EAPC(95%UI) 1990-2021 |
| --- | --- | --- | --- | --- | --- |
|  | Number (95% UI) | ASR (95% UI) | Number (95% UI) | ASR (95% UI) |  |
| Global | 5367136.581 (5076403.864,5562773.874) | 158.9 (148.141,165.296) | 8991636.684 (8264123.214,9531130.166) | 108.728 (99.597,115.382) | -1.301 (-1.343,-1.258) |
| **SDI quintiles** |  |  |  |  |  |
| High SDI | 1732392.148 (1591274.947,1797849.331) | 157.59 (144.187,163.925) | 1392371.048 (1217171.556,1489069.661) | 58.451 (52.18,61.924) | -3.428 (-3.537,-3.319) |
| High-middle SDI | 1604862.169 (1527808.263,1657939.758) | 193.936 (182.106,200.601) | 2450426.347 (2218836.447,2647300.493) | 127.495 (115.029,137.722) | -1.564 (-1.779,-1.348) |
| Middle SDI | 1038968.247 (978516.151,1100277.108) | 127.055 (118.353,134.973) | 2811121.62 (2564657.442,3023433.626) | 118.714 (107.231,127.805) | -0.115 (-0.189,-0.042) |
| Low SDI | 228811.487 (202386.134,255182.859) | 119.502 (106.259,132.453) | 493475.838 (448128.795,543115.165) | 116.413 (105.212,127.692) | 0.006 (-0.106,0.118) |
| Low-middle SDI | 753129.845 (698697.089,804552.237) | 140.99 (129.758,151.24) | 1834652.91 (1699506.178,1964078.891) | 142.098 (131.304,151.866) | 0.159 (0.083,0.235) |
| **GBD regions** |  |  |  |  |  |
| Andean Latin America | 17089.417 (15548.218,18721.244) | 92.947 (84.558,101.238) | 32964.663 (28040.242,39366.223) | 58.174 (49.561,69.332) | -1.741 (-2.134,-1.347) |
| Australasia | 40160.879 (37288.991,41659.239) | 176.763 (162.817,184.023) | 28640.381 (24369.918,30972.532) | 46.672 (40.229,50.211) | -4.524 (-4.626,-4.422) |
| Caribbean | 44603.13 (42479.944,46171.144) | 188.967 (179.079,195.789) | 61449.989 (54942.166,69041.027) | 112.503 (100.506,126.541) | -1.706 (-1.907,-1.505) |
| Central Asia | 131771.206 (124692.116,136248.92) | 320.468 (299.831,332.361) | 175394.031 (159202.722,192406.549) | 265.508 (240.67,290.416) | -1.012 (-1.299,-0.724) |
| Central Europe | 364089.926 (349914.689,371867.897) | 272.563 (259.221,279.183) | 331281.294 (299877.575,352596.606) | 139.983 (126.838,148.911) | -2.455 (-2.562,-2.349) |
| Central Latin America | 88861.76 (84857.222,90852.223) | 125.723 (118.689,129.144) | 247050.822 (221193.823,273150.299) | 103.686 (92.476,114.586) | -0.764 (-1.006,-0.522) |
| Central Sub-Saharan Africa | 23271.654 (18250.79,29475.763) | 134.901 (107.86,167.196) | 49849.974 (38888.576,63650.142) | 119.337 (93.699,150.292) | -0.572 (-0.659,-0.485) |
| East Asia | 570428.711 (505994.31,639930.43) | 93.924 (83.871,105.304) | 2008011.056 (1683969.688,2335232.271) | 108.903 (91.175,125.787) | 0.896 (0.573,1.221) |
| Eastern Europe | 786270.579 (751128.306,803303.11) | 323.174 (305.258,331.759) | 903624.608 (811062.832,990580.965) | 252.887 (226.965,277.152) | -1.237 (-1.71,-0.762) |
| Eastern Sub-Saharan Africa | 43989.28 (39100.715,50522.157) | 69.438 (61.048,78.851) | 101222.405 (87734.685,117265.404) | 72.16 (62.092,82.994) | -0.055 (-0.144,0.034) |
| High-income Asia Pacific | 117621.576 (107063.627,122952.524) | 67.038 (59.851,70.504) | 152702.311 (123996.014,168788.529) | 25.564 (21.776,27.645) | -3.041 (-3.208,-2.874) |
| High-income North America | 644903.976 (580448.049,675667.315) | 177.725 (160.274,186.075) | 534770.385 (468399.901,571529.316) | 75.849 (67.172,80.598) | -3.09 (-3.244,-2.936) |
| North Africa and Middle East | 386036.533 (357948.996,418208.393) | 275.176 (253.616,299.125) | 769135.138 (685360.014,858253.159) | 202.849 (180.591,223.679) | -1.026 (-1.077,-0.975) |
| Oceania | 4528.185 (3745.938,5489.131) | 182.546 (155.474,217.424) | 11136.859 (9328.753,13321.115) | 170.887 (145.426,201.151) | -0.182 (-0.226,-0.139) |
| South Asia | 708220.977 (643038.475,771051.831) | 136.388 (122.875,149.554) | 1990113.467 (1824490.292,2155696.108) | 149.137 (136.973,161.164) | 0.436 (0.296,0.576) |
| Southeast Asia | 252646.985 (229370.468,275073.755) | 114.719 (103.393,125.652) | 638703.889 (575906.562,694079.451) | 110.916 (100.177,120.203) | -0.103 (-0.184,-0.022) |
| Southern Latin America | 62788.281 (59932.981,64743.188) | 149.426 (141.12,154.542) | 49098.502 (45022.285,51734.533) | 54.407 (50.085,57.26) | -2.918 (-3.083,-2.754) |
| Southern Sub-Saharan Africa | 17901.165 (15561.876,19842.891) | 75.959 (64.948,84.841) | 39816.576 (36778.001,43187.111) | 83.44 (76.934,90.186) | 0.266 (-0.145,0.678) |
| Tropical Latin America | 107545.377 (102011.548,110638.536) | 135.908 (126.395,140.82) | 162299.204 (149052.806,170324.773) | 64.487 (58.976,67.843) | -2.272 (-2.371,-2.172) |
| Western Europe | 879289.865 (811263.829,910871.037) | 148.222 (136.541,153.847) | 543038.415 (463990.182,584339.4) | 47.27 (41.445,50.419) | -3.894 (-3.995,-3.793) |
| Western Sub-Saharan Africa | 75117.12 (64540.842,87128.792) | 105.292 (90.241,121.557) | 161332.716 (140025.901,185440.451) | 105.971 (92.827,120.168) | 0.024 (-0.106,0.155) |
