# Supplementary figures and images for: Trends and predictive research on the global burden of ischemic heart disease from 1990 to 2021: an analysis of the Global Burden of Disease study 2021
Source: Front Public Health. 2025 Sep 19;13:1569179. doi: 10.3389/fpubh.2025.1569179 (PMC12491020; doi:10.3389/fpubh.2025.1569179)

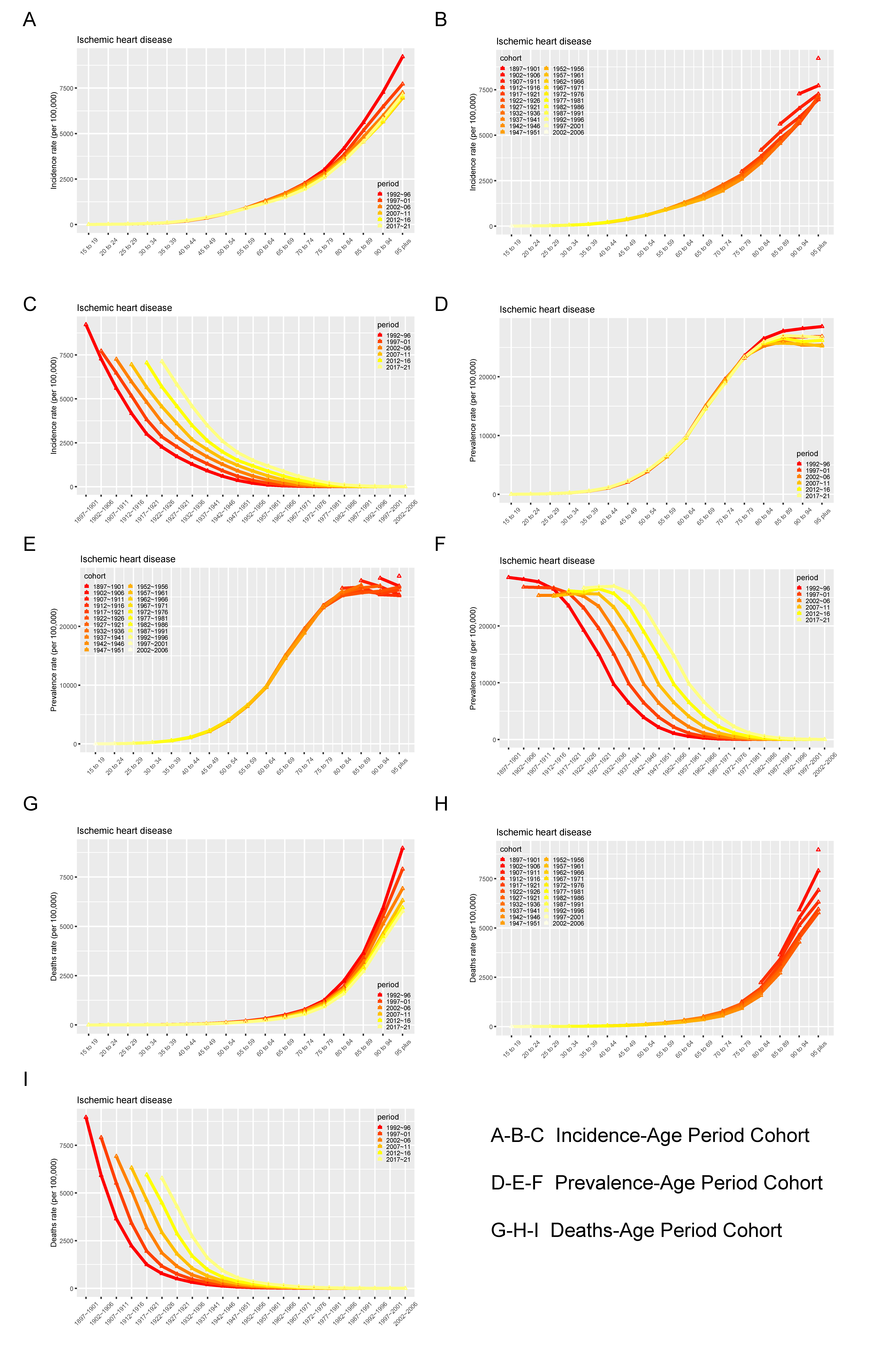

Supplement: Supplementary file 15 [file Image_1.tif]
